# A multinational Delphi consensus to end the COVID-19 public health threat

**DOI:** 10.1038/s41586-022-05398-2

**Published:** 2022-11-03

**Authors:** Jeffrey V. Lazarus, Diana Romero, Christopher J. Kopka, Salim Abdool Karim, Laith J. Abu-Raddad, Gisele Almeida, Ricardo Baptista-Leite, Joshua A. Barocas, Mauricio L. Barreto, Yaneer Bar-Yam, Quique Bassat, Carolina Batista, Morgan Bazilian, Shu-Ti Chiou, Carlos del Rio, Gregory J. Dore, George F. Gao, Lawrence O. Gostin, Margaret Hellard, Jose L. Jimenez, Gagandeep Kang, Nancy Lee, Mojca Matičič, Martin McKee, Sabin Nsanzimana, Miquel Oliu-Barton, Bary Pradelski, Oksana Pyzik, Kenneth Rabin, Sunil Raina, Sabina Faiz Rashid, Magdalena Rathe, Rocio Saenz, Sudhvir Singh, Malene Trock-Hempler, Sonia Villapol, Peiling Yap, Agnes Binagwaho, Adeeba Kamarulzaman, Ayman El-Mohandes, The COVID-19 Consensus Statement Panel, Mauricio Barreto, Carlos del Rio, Salim Abdulla, Sarah Addleman, Gulnara Aghayeva, Raymond Agius, Mohammed Ahmed, Mohamed Ahmed Ramy, Pedro Aide, Soo Aleman, Jean-Patrick Alfred, Shamim Ali, Jorge Aliaga, Tammam Aloudat, Saleh A. Alqahtani, Jameela Al-Salman, John H. Amuasi, Anurag Agrawal, Wagida Anwar, Tania Araujo-Jorge, Osvaldo Artaza, Leyla Asadi, Yaw Awuku, Michael Baker, Lorena Barberia, Ernesto Bascolo, Paul Belcher, Lizett Bell, Adele Benzaken, Emil Bergholtz, Nahid Bhadelia, Anant Bhan, Stephane Bilodeau, Ricardo Bitrán, Philomena Bluyssen, Arnold Bosman, Fernando A. Bozza, Melanie M. Brinkmann, Andrew Brown, Bruce Mellado, Elizabeth Bukusi, Chris Bullen, Giorgio Buonanno, Rochelle Burgess, Matthew Butler, Pauline Byakika-Kibwika, Baltica Cabieses, Gunilla Carlsson, Fidelia Cascini, Chishala Chabala, Mohamed Chakroun, K. K. Cheng, Agnes Chetty, Dmytro Chumachenko, Gregg Consalves, Andrew Conway Morris, Ahmed Cordie, Tumani Corrah, Brenda Crabtree-Ramírez, Naranjargal Dashdorj, Nadav Davidovitch, Luis Eugenio de Souza, Akshay Chand Dhariwal, Elena Druică, Onder Ergonul, Ngozi A. Erondu, Mohammad Yasir Essar, Andrew Ewing, Gonzalo Fanjul, Daniel Feierstein, Eric Feigl-Ding, Ramon Figueroa, John Peter Figueroa, Dale Fisher, Walter Flores, David A. Forero-Peña, Howard Frumkin, Amiran Gamkrelidze, Monica Gandhi, Patricia Garcia, Alberto L. Garcia-Basteiro, Adolfo García-Sastre, Suneela Garg, F. A. Gbeasor-Komlanvi, Carlos Gershenson, Ishwar Gilada, Ligia Giovanella, Marino González, Manfred S. Green, Trisha Greenhalgh, Paul Griffin, Stephen Griffin, Beatriz Grinsztejn, Tanu Anand, Germán Guerra, Renzo Guinto, Mariusz Gujski, Rahmet Guner, Adam Hamdy, Marian-Gabriel Hâncean, Abusayeed Haniffa, Kenneth Y. Hartigan-Go, Hoda K. Hassan, Simon I. Hay, Matti T. J. Heino, Zdenek Hel, Peter Hotez, Jia Hu, Mirsada Hukić, Carel IJsselmuiden, Davidson Iroko, Maged Iskarous, Chimaraoke Izugbara, Choolwe Jacobs, Alejandro R. Jadad, Fyezah Jehan, Ayana Jordan, Imane Jroundi, Kevin Kain, Fatjona Kamberi, Eduard Karamov, Abraar Karan, Rebecca Katz, Aris Katzourakis, Abigail Kazembe, Faryal Khamis, Komiljon Khamzayev, Judy Khanyola, Kamlesh Khunti, Elsie Kiguli-Malwadde, Woo Joo Kim, Bruce J. Kirenga, Daniel Klimovský, Brittany L. Kmush, Felicia Knaul, Manolis Kogevinas, Frederik Kristensen, Dinesh Kumar, Raman Kumar, Amanda Kvalsvig, Marcus V. Lacerda, Arush Lal, Tom Lawton, Jay Lemery, Anthony J. Leonardi, Yuguo Li, Jan Löttvall, Mohamed Lounis, Daniel Maceira, C. Raina MacIntyre, Azzeddine Madani, Gkikas Magiorkinis, Reza Malekzadeh, Marc Choisy, Jasmine R. Marcelin, Guy B. Marks, Linsey Marr, Jeanne Marrazzo, Antonieta Martina, José M. Martín-Moreno, Carlos Mateos, Mayfong Mayxay, Jean Bapiste Mazarati, Souleymane Mboup, Jennifer McDonald, Faye McMillan, Enkeleint Mechili, Andre Medici, Sarah L. M. Davis, Petra Meier, Ziad A. Memish, Jaideep Menon, Purnima Menon, Jonathan Mesiano-Crookston, Susan Michie, Ivana Mikolasevic, Ognjen Milicevic, Asit Kumar Mishra, Rahma Mohamed, Ali H. Mokdad, Michele Monroy-Valle, Lidia Morawska, Sterghios A. Moschos, Karam Motawea, Sayed Hamid Mousavi, Ghina Mumtaz, Peter K. Munene, Carmen Muñoz Almagro, Janet Muriuki, Sylvia Muyingo, Denise Naniche, C. David Naylor, Nicaise Ndembi, Juraj Nemec, Igor Nesteruk, Christine Ngaruiya, Hung Nguyen, Dafina Nikolova, Dorit Nitzan, Ole Norheim, Mohammed Noushad, Francine Ntoumi, Gunhild Alvik Nyborg, Eleanor Ochodo, Zekaver Odabasi, Mbah Patrick Okwen, Keiser Olivia, David S. Y. Ong, Ijeoma Opara, Miguel Orozco, Hitoshi Oshitani, Christina Pagel, Madhukar Pai, Björg Pálsdóttir, Georgios Papatheodoridis, Dimitrios Paraskevis, Jeanna Parsons Leigh, Bernard Pécoul, Andreas Peichl, Eddy Perez-Then, Phuc Pham Duc, Cécile Philippe, Andrea Pineda Rojas, Courtney Pladsen, Anton Pozniak, Rodrigo Quiroga, Huma Qureshi, Sanjay Rampal, Megan Ranney, Laura Rathe, Scott Ratzan, Henriette Raventos, Helen Rees, Renata Reis, Walter Ricciardi, Nesrine Rizk, Magda Robalo, Eleanor Robertson, Leanne Robinson, Casper Rokx, Tamsin Ros, John-Arne Røttingen, Meir Rubin, Kiat Ruxrungtam, Shakhlo Sadirova, Senjuti Saha, Nelly Salgado, Lizet Sanchez, Thurka Sangaramoorthy, Carolina Santamaria-Ulloa, Renata Santos, Bisher Sawaf, Matthias F. Schneider, Robert T. Schooley, Alper Sener, Jaime Sepulveda, Jaffer Shah, Mosa Shibani, Sheikh Shoib, Izukanji Sikazwe, Aistis Šimaitis, Amandeep Singh Gill, Natia Skhvitaridze, Milka Sokolović, Roma Solomon, Xavier Solórzano, Sandra A. Springer, Jakub Šrol, Anthony Staines, Henry T. Stelfox, Steffanie Strathdee, Lokman Hakim Sulaiman, Brett Sutton, Dag Svanæs, Sarya Swed, Vana Sypsa, Kristine Sørensen, Raji Tajudeen, Amy Tan, Julian Tang, Marcel Tanner, Tavpritesh Sethi, Marleen Temmerman, Kyu Kyu Than, Halidou Tinto, Sênoudé Pacôme Tomètissi, Irene Torres, K. P. Tshering, Sotirios Tsiodras, Benjamin Tsofa, Anders Vahlne, Juan Rafael Vargas, Ivan Dario Velez Bernal, Deisy Ventura, Rafael Vilasanjuan, Joe Vipond, Sarah Wamala-Andersson, Pawel Wargocki, Robert West, Angela Weyand, Trenton M. White, Guntram Wolff, Maosheng Yao, Christian A. Yates, Georgina Yeboah, Leo Yee-Sin, Siyan Yi, Yik-Ying Teo, Poovorawan Yong, Victor Zamora-Mesía, Anne Øvrehus

**Affiliations:** 1 Barcelona Institute for Global Health (ISGlobal), Barcelona, Spain; 2 Faculty of Medicine and Health Sciences, University of Barcelona, Barcelona, Spain; 3 City University of New York Graduate School of Public Health and Health Policy (CUNY SPH), New York City, NY USA; 4 Independent Researcher, Sioux Falls, SD USA; 5 University of KwaZulu-Natal, Durban, South Africa; 6 Centre for the AIDS Program of Research in South Africa (CAPRISA), Durban, South Africa; 7 Weill Cornell Medicine, Cornell University, Ithaca, NY USA; 8 Weill Cornell Medicine-Qatar, Cornell University, Qatar Foundation-Education City, Doha, Qatar; 9 Pan American Health Organisation, Washington, DC USA; 10 UNITE Global Parliamentarians Network, Lisbon, Portugal; 11 Faculty of Health, Medicine and Life Sciences, Maastricht University, Maastricht, The Netherlands; 12 Institute of Health Sciences (CIIS), Catholic University of Portugal, Lisbon, Portugal; 13 University of Colorado School of Medicine, Aurora, CO USA; 14 Oswaldo Cruz Foundation (Fiocruz), Rio de Janeiro, Brazil; 15 University of Bahia, Salvador, Brazil; 16 New England Complex Systems Institute, Cambridge, MA USA; 17 Manhiça Health Research Center (CISM), Maputo, Mozambique; 18 Catalan Institute for Research and Advanced Studies (ICREA), Barcelona, Spain; 19 Pediatrics Department, Hospital Sant Joan de Déu, University of Barcelona, Barcelona, Spain; 20 Biomedical Research Consortium in Epidemiology and Public Health (CIBERESP), Madrid, Spain; 21 Doctors Without Borders (MSF), Geneva, Switzerland; 22 Baraka Impact Finance, Geneva, Switzerland; 23 Payne Institute, Colorado School of Mines, Golden, CO USA; 24 National Yang Ming Chiao Tung University, Taipei, Taiwan; 25 Emory School of Medicine, Atlanta, GA USA; 26 University of New South Wales (UNSW) Sydney, Sydney, New South Wales Australia; 27 Chinese Center for Disease Control and Prevention, Beijing, China; 28 The O’Neill Institute for National and Global Health Law, Georgetown University, Washington, DC USA; 29 Burnet Institute, Melbourne, Victoria Australia; 30 Department of Chemistry, University of Colorado Boulder, Boulder, CO USA; 31 Cooperative Institute for Research in Environmental Sciences (CIRES), University of Colorado Boulder, Boulder, CO USA; 32 Christian Medical College (CMC), Vellore, India; 33 Wilton Park, Steyning, UK; 34 Clinic for Infectious Diseases and Febrile Illnesses, University Medical Centre, Ljubljana, Slovenia; 35 Faculty of Medicine, University of Ljubljana, Ljubljana, Slovenia; 36 The London School of Hygiene & Tropical Medicine, London, UK; 37 University Teaching Hospital of Butare, Butare, Rwanda; 38 Paris Dauphine University - PSL, Paris, France; 39 French National Centre for Scientific Research (CNRS), Grenoble, France; 40 University College London (UCL), London, UK; 41 Dr. Rajendra Prasad Government Medical College, Himachal Pradesh, India; 42 James P. Grant School of Public Health, BRAC University, Dhaka, Bangladesh; 43 Plenitud Foundation, Santo Domingo, Dominican Republic; 44 University of Costa Rica, San José, Costa Rica; 45 Faculty of Medical and Health Sciences, University of Auckland, Auckland, New Zealand; 46 Independent Philosopher, Copenhagen, Denmark; 47 Department of Neurosurgery, Houston Methodist Research Institute, Houston, TX USA; 48 International Digital Health & AI Research Collaborative (I-DAIR), Geneva, Switzerland; 49 University of Global Health Equity, Kigali, Rwanda; 50 University of Malaya, Kuala Lumpur, Malaysia; 51 Ifakara Health Institute, Dar es Salaam, Tanzania; 52 University of Ottawa, Ottawa, Ontario Canada; 53 Baku Health Centre, Baku, Azerbaijan; 54 The University of Manchester, Manchester, UK; 55 Mogadishu University, Mogadishu, Somalia; 56 Generations for Health, Madrid, Spain; 57 Karolinska University Hospital, Stockholm, Sweden; 58 Ministry of Health and Population, Port-au-Prince, Haiti; 59 Moi University, Eldoret, Kenya; 60 Hurlingham National University, Hurlingham, Argentina; 61 Graduate Institute of International and Development Studies, Geneva, Switzerland; 62 King Faisal Specialist Hospital and Research Centre, Riyadh, Saudi Arabia; 63 Salmaniya Medical Complex, Manama, Bahrain; 64 Kwame Nkrumah University of Science and Technology, Kumasi, Ghana; 65 Trivedi School of Biosciences, Ashoka University, Sonepat, India; 66 Community Medicine Department, Faculty of Medicine, Ain Shams University, Cairo, Egypt; 67 Faculty of Health and Social Sciences, University of The Americas, Santiago, Chile; 68 University of Alberta, Edmonton, Alberta Canada; 69 University of Health and Allied Sciences, Ho, Volta Ghana; 70 University of Otago, Wellington, New Zealand; 71 University of Sao Paulo, Sao Paulo, Brazil; 72 European Public Health Alliance, Brussels, Belgium; 73 Ministry of Health and Wellness Belize, Belmopan, Belize; 74 Aids Healthcare Foundation, São Paulo, Brazil; 75 Department of Physics, Stockholm University, Stockholm, Sweden; 76 Boston University Center for Emerging Infectious Diseases Policy and Research (CEID), Boston, MA USA; 77 Centre For Ethics, Yenepoya University, Mangaluru, India; 78 Smart Phases, Plattsburgh, NY USA; 79 Bitran y Asociados, Santiago, Chile; 80 Delft University of Technology, Delft, The Netherlands; 81 Transmissible, Houten, The Netherlands; 82 Institute of Genetics, Technische Universität Braunschweig, Braunschweig, Germany; 83 Management Sciences for Health, Medford, MA USA; 84 University of the Witwatersrand, Johannesburg, South Africa; 85 Kenya Medical Research Institute, Nairobi, Kenya; 86 University of Cassino and Southern Lazio, Cassino, Italy; 87 King’s College London, London, UK; 88 Department of Medicine, Makerere University College of Health Sciences, Kampala, Uganda; 89 Universidad del Desarrollo, Santiago, Chile; 90 The Global Fund, Geneva, Switzerland; 91 Catholic University of the Sacred Heart, Rome, Italy; 92 University of Zambia, Lusaka, Zambia; 93 Fattouma Bourguiba Teaching Hospital, Monastir, Tunisia; 94 Institute of Applied Health Research, University of Birmingham, Birmingham, UK; 95 Ministry of Health Seychelles, Victoria, Seychelles; 96 National Aerospace University “Kharkiv Aviation Institute”, Kharkiv, Ukraine; 97 Yale School of Public Health, New Haven, CT USA; 98 University of Cambridge, Cambridge, UK; 99 Cairo University Hospitals, Cairo, Egypt; 100 Africa Research Excellence Fund (AREF), London, UK; 101 Instituto Nacional de Ciencias Médicas y Nutrición Salvador Zubirán, Mexico City, Mexico; 102 Onom Foundation, Ulaanbaatar, Mongolia; 103 Ben Gurion University of the Negev, Be’er Sheva, Israel; 104 National Centre for Disease Control and National Vector Borne Disease Control Programme, Government of India, Delhi, India; 105 University of Bucharest, Bucharest, Romania; 106 Koç University İşbank Center for Infectious Diseases, Istanbul, Turkey; 107 Kabul University of Medical Sciences, Kabul, Afghanistan; 108 University of Gothenburg, Gothenburg, Sweden; 109 CONICET/National University of Tres de Febrero, Caseros, Argentina; 110 Federation of American Scientists, Washington, DC USA; 111 Social Security Board, Belize City, Belize; 112 The University of the West Indies, Kingston, Jamaica; 113 National University of Singapore, Singapore, Singapore; 114 Center for the Study of Equity and Governance in Health Systems, Guatemala City, Guatemala; 115 Biomedical Research and Therapeutic Vaccines Institute, Ciudad Bolivar, Venezuela; 116 Department of Health Metrics Sciences, University of Washington, Seattle, WA USA; 117 National Center for Disease Control and Public Health of Georgia, Tbilisi, Georgia; 118 UCSF, San Francisco, CA USA; 119 School of Public Health, Cayetano Heredia University, Lima, Peru; 120 Icahn School of Medicine at Mount Sinai, New York City, NY USA; 121 MAMC & Associated Hospitals New Delhi, New Delhi, India; 122 University of Lomé, Lomé, Togo; 123 Universidad Nacional Autonoma de Mexico, Mexico City, Mexico; 124 Organised Medicine Academic Guild—OMAG, Mumbai, India; 125 Simon Bolivar University, Caracas, Venezuela; 126 University of Haifa, Haifa, Israel; 127 University of Oxford, Oxford, UK; 128 University of Queensland, Brisbane, Queensland Australia; 129 University of Leeds, Leeds, UK; 130 National Institute of Infectology Evandro Chagas-Fiocruz, Rio de Janeiro, Brazil; 131 Indian Council of Medical Research, New Delhi, India; 132 National Institute of Public Health, Cuernavaca, Mexico; 133 St Luke’s Medical Center College of Medicine, Quezon City, The Philippines; 134 Department of Public Health, Medical University of Warsaw, Warsaw, Poland; 135 Ankara City Hospital Infectious Diseases and Clinical Mirobiology, Ankara, Turkey; 136 Independent Researcher, Port Louis, Mauritius; 137 Ministry of Health, Colombo, Sri Lanka; 138 Ateneo School of Government, Quezon City, The Philippines; 139 Independent Consultant, Cairo, Egypt; 140 University of Helsinki, Helsinki, Finland; 141 University of Alabama at Birmingham, Birmingham, AL USA; 142 National School of Tropical Medicine, Baylor College of Medicine, Houston, TX USA; 143 19 To Zero, Calgary, Alberta Canada; 144 Center for Disease Control and Geohealth Studies, Academy of Sciences and Arts of Bosnia and Herzegovina, Sarajevo, Bosnia and Herzegovina; 145 COHRED, Geneva, Switzerland; 146 Independent Consultant, Cairo, Egypt; 147 International Center for Research on Women, Washington, DC USA; 148 Centre for Digital Therapeutics, Toronto, Ontario Canada; 149 Aga Khan University, Karachi, Pakistan; 150 NYU Grossman School of Medicine, New York City, NY USA; 151 School of Medicine and Pharmacy, University Mohammed V, Rabat, Morocco; 152 University of Toronto, Toronto, Ontario Canada; 153 Research Centre of Public Health, Faculty of Health, University of Vlore “Ismail Qemali” Albania, Vlorë, Albania; 154 Gamaleya National Research Center for Epidemiology and Microbiology of the Ministry of Health of the Russian Federation, Moscow, Russian Federation; 155 Division of Infectious Disease & Geographic Medicine, Stanford University, Stanford, CA USA; 156 Kamuzu University of Health Sciences, Blantyre, Malawi; 157 Royal Hospital, Ministry of Health, Muscat, Oman; 158 Tashkent Pediatric Medical Institute, Tashkent, Uzbekistan; 159 University of Leicester, Leicester, UK; 160 African Center for Global Health and Social Transformation, Kampala, Uganda; 161 Division of Infectious Diseases, Guro Hospital, College of Medicine Korea University, Seoul, Republic of Korea; 162 Comenius University in Bratislava, Bratislava, Slovakia; 163 Syracuse University, Syracuse, NY USA; 164 University of Miami Institute for Advanced Study of the Americas, Coral Gables, FL USA; 165 Coalition of Epidemic Preparedness Innovations (CEPI), Oslo, Norway; 166 Institute of Family Medicine and Primary Care, Greater Noida, India; 167 London School of Economics & Political Science, London, UK; 168 Bradford Institute for Health Research & Bradford Teaching Hospitals NHS Foundation Trust, Bradford, UK; 169 St Georges University of London, London, UK; 170 The University of Hong Kong, Hong Kong, China; 171 University Ziane Achour Djelfa, Djelfa, Algeria; 172 University of Buenos Aires/Health Systems Global, Buenos Aires, Argentina; 173 Faculty of Social and Human Sciences, Khemis-Miliana University, Khemis Miliana, Algeria; 174 National and Kapodistrian University of Athens, Athens, Greece; 175 Digestive Disease Research Institute Tehran University of Medical Sciences, Tehran, Iran; 176 Centre for Tropical Medicine and Global Health, Nuffield Department of Medicine, University of Oxford, Oxford, UK; 177 University of Nebraska Medical Center, Omaha, NE USA; 178 Virginia Tech, Blacksburg, VA USA; 179 University of Cabo Verde, Praia, Cape Verde; 180 Department of Preventive Medicine and Public Health and INCLIVA, University of Valencia, Valencia, Spain; 181 Instituto #SaludsinBulos, Madrid, Spain; 182 University of Health Sciences, Ministry of Health, Vientiane, Lao People’s Democratic Republic; 183 Foundation for Innovative New Diagnostics, Geneva, Switzerland; 184 Institute of Health Research Epidemiological Surveillance and Trainings (IRESSEF), Dakar, Senegal; 185 Universal Health Monitor, Bethesda, MD USA; 186 University of Glasgow, Glasgow, UK; 187 King Saud Medical City, Ministry of Health and College of Medicine Alfaisal University, Riyadh, Saudi Arabia; 188 Amrita Institute of Medical Sciences, Kochi, India; 189 International Food Policy Research Institute, New Delhi, India; 190 Goldman Hine LLP, Toronto, Ontario Canada; 191 Department of Gastroeterology, University Hospital Center Riejka, Riejka, Croatia; 192 School of Medicine, University of Belgrade, Belgrade, Serbia; 193 MaREI Centre, Ryan Institute & School of Engineering, University of Galway, Galway, Ireland; 194 University of Saskatchewan, Saskatoon, Saskatchewan Canada; 195 Queensland University of Technolgy, Brisbane, Queensland Australia; 196 Department of Applied Sciences, Faculty of Health and Life Sciences, Northumbria University, Newcastle, UK; 197 Faculty of Medicine, Alexandria University, Alexandria, Egypt; 198 New York State Department of Health, Albany, NY USA; 199 American University of Beirut, Beirut, Lebanon; 200 Faith to Action Network, Nairobi, Kenya; 201 International University of Catalonia, Barcelona, Spain; 202 IntraHealth International, Nairobi, Kenya; 203 African Population and Health Research Center, Nairobi, Kenya; 204 Africa Centers for Disease Control and Prevention (Africa CDC), Addis Ababa, Ethiopia; 205 Masaryk University, Brno, Czech Republic; 206 Institute of Hydromechanics, National Academy of Sciences of Ukraine, Kiev, Ukraine; 207 Yale School of Medicine, New Haven, CT USA; 208 International Livestock Research Institute, Nairobi, Kenya; 209 University Clinic of Gastroenterohepatology, Faculty of Medicine, University “Ss. Cyril and Methodius”, Skopje, Republic of Macedonia; 210 University of Bergen, Bergen, Norway; 211 College of Dentistry, Dar Al Uloom University, Riyadh, Saudi Arabia; 212 Congolese Foundation for Medical Research, Brazzaville, Republic of the Congo; 213 Institute of Tropical Medicine, University of Tübingen, Tübingen, Germany; 214 Covid Action Group, World Health Network, Son, Norway; 215 Department of Infectious Diseases, Internal Medicine, School of Medicine, Marmara University, Istanbul, Turkey; 216 Effective Basic Services (eBASE) Africa, Bamenda, Cameroon; 217 University of Geneva, Institute of Global Health, Geneva, Switzerland; 218 Franciscus Gasthuis en Vlietland, Rotterdam, The Netherlands; 219 Wayne State University School of Medicine, Detroit, MI USA; 220 International Independent Expert on Health Systems, Managua, Nicaragua; 221 Tohoku University Graduate School of Medicine, Sendai, Japan; 222 McGill School of Population and Global Health, Montreal, Quebec Canada; 223 Training for Health Equity Network: THEnet, New York City, NY USA; 224 Dalhousie University, Halifax, Nova Scotia Canada; 225 Drugs for Neglected Diseases initiative, Geneva, Switzerland; 226 ifo Institute at the University of Munich, Munich, Germany; 227 Two Oceans in Health, Santo Domingo, Dominican Republic; 228 Vietnam One Health University Network (VOHUN), Hanoi University of Public Health (HUPH), Hanoi, Vietnam; 229 Molinari Economic Institute, Paris, France; 230 CIMA, UMI-IFAECI/CNRS, FCEyN, Universidad de Buenos Aires-UBA/CONICET, Buenos Aires, Argentina; 231 National Health Care for the Homeless Council, Nashville, TN USA; 232 Chelsea and Westminster Hospital, London, UK; 233 INFIQC-CONICET, National University of Córdoba, Córdoba, Argentina; 234 Department of Botany, University of Chakwal, Chakwal, Pakistan; 235 Brown University, Providence, RI USA; 236 Institute for Global Health and Development, Bissau, Guinea-Bissau; 237 NHS Greater Glasgow and Clyde, Glasgow, UK; 238 Erasmus University Medical Center, Rotterdam, The Netherlands; 239 Africa Europe Foundation, Brussels, Belgium; 240 Norwegian Institute of Public Health, Oslo, Norway; 241 Kohelet Policy Forum, Jerusalem, Israel; 242 Chulalongkorn University, Bangkok, Thailand; 243 Research Institute of Virology under the Ministry of Health of Uzbekistan, Tashkent, Uzbekistan; 244 Child Health Research Foundation, Dhaka, Bangladesh; 245 National Institute of Public Health, Mexico City, Mexico; 246 Center of Molecular Immunology, La Havana, Cuba; 247 University of Maryland, College Park, MD USA; 248 Internal Medicine Department, Hamad Medical Corporation, Doha, Qatar; 249 Technical University of Dortmund, Dortmund, Germany; 250 University of California San Diego, San Diego, CA USA; 251 Infectious Disease Department, Izmir Katip Celebi University Ataturk Training and Research Hospital, Izmir, Turkey; 252 Faculty of Medicine, Syrian Private University, Damascus, Syrian Arab Republic; 253 Department of Psychiatry, Jawahar Lal Nehru Memorial Hospital, Srinagar, India; 254 Centre for Infectious Disease Research in Zambia (CIDRZ), Lusaka, Zambia; 255 The Office of the Government of the Republic of Lithuania, Vilnius, Lithuania; 256 CORE Group Polio Project India, Delhi, India; 257 Independent Consultant, Quito, Ecuador; 258 Institute of Experimental Psychology, Centre of Social and Psychological Sciences, Slovak Academy of Sciences, Bratislava, Slovakia; 259 Dublin City University, Dublin, Ireland; 260 University of Calgary, Calgary, Alberta Canada; 261 Institute for Research, Development and Innovation, International Medical University, Kuala Lumpur, Malaysia; 262 Department of Health, Melbourne, Victoria Australia; 263 Norwegian University of Science and Technology, Trondheim, Norway; 264 Faculty of Medicine, Aleppo University, Aleppo, Syrian Arab Republic; 265 Global Health Literacy Academy, Risskov, Denmark; 266 African Union, Addis Ababa, Ethiopia; 267 University of British Columbia, Vancouver, British Columbia Canada; 268 Swiss Academies of Arts and Sciences, Bern, Switzerland; 269 Indraprastha Institute of Information Technology, Delhi, India; 270 Centre of Excellence in Women and Child Health, Aga Khan University, Nairobi, Kenya; 271 Burnet Institute Myanmar, Yangon, Myanmar; 272 Institute for Health Sciences Research (CNRST/IRSS), Nanoro, Burkina Faso; 273 People’s Health Movement, Abomey-Calavi, Benin; 274 Fundacion Octaedro, Quito, Ecuador; 275 Khesar Gyalpo University of Medical Sciences of Bhutan, Thimphu, Bhutan; 276 Antioquia University, Medellin, Colombia; 277 Mälardalens University, Mälardalens, Sweden; 278 Technical University of Denmark, Kongens Lyngby, Denmark; 279 University of Michigan Medical School, Ann Arbor, MI USA; 280 Hospital Clínic, University of Barcelona, Barcelona, Spain; 281 Bruegel Free University of Brussels, Brussels, Belgium; 282 College of Environmental Sciences and Engineering, Peking University, Beijing, China; 283 University of Bath, Bath, UK; 284 African Forum for Research and Education in Health, Kumasi, Ghana; 285 National Centre for Infectious Diseases, Singapore, Singapore; 286 Gobierna Consulting Firm, Lima, Peru; 287 Department of Infectious Diseases, Odense University Hospital, Odense, Denmark

**Keywords:** Public health, Policy, Health policy

## Abstract

Despite notable scientific and medical advances, broader political, socioeconomic and behavioural factors continue to undercut the response to the COVID-19 pandemic^1,2^. Here we convened, as part of this Delphi study, a diverse, multidisciplinary panel of 386 academic, health, non-governmental organization, government and other experts in COVID-19 response from 112 countries and territories to recommend specific actions to end this persistent global threat to public health. The panel developed a set of 41 consensus statements and 57 recommendations to governments, health systems, industry and other key stakeholders across six domains: communication; health systems; vaccination; prevention; treatment and care; and inequities. In the wake of nearly three years of fragmented global and national responses, it is instructive to note that three of the highest-ranked recommendations call for the adoption of whole-of-society and whole-of-government approaches^1^, while maintaining proven prevention measures using a vaccines-plus approach^2^ that employs a range of public health and financial support measures to complement vaccination. Other recommendations with at least 99% combined agreement advise governments and other stakeholders to improve communication, rebuild public trust and engage communities^3^ in the management of pandemic responses. The findings of the study, which have been further endorsed by 184 organizations globally, include points of unanimous agreement, as well as six recommendations with >5% disagreement, that provide health and social policy actions to address inadequacies in the pandemic response and help to bring this public health threat to an end.

## Abstract (web summary)

A diverse, multidisciplinary panel of 386 experts in COVID-19 response from 112 countries provides health and social policy actions to address inadequacies in the pandemic response and help to bring this public health threat to an end.

## Main

Pandemics have disrupted societies and impacted public health throughout human history^4^. Today, almost 3 years after SARS-CoV-2 was first identified and more than 1.5 years after the first vaccines became available, pandemic fatigue^5^ threatens to undercut our vigilance and the effectiveness of our responses to ongoing and new pandemic-related challenges. As of September 2022, more than 620 million cases of COVID-19 and over 6.5 million deaths have been reported^6^, although mortality estimates range as high as 20 million^7,8^. The healthcare for millions more people has been delayed, often as a result of overwhelmed health systems^9–12^. Highly transmissible variants continue to spread globally, while surveillance for variants of concern remains largely inadequate^13–15^. Reinfection risks are not fully understood. Low vaccination rates^16^ may compound the risk from waning immunity^17,18^. Long COVID has emerged as a serious chronic condition^19–21^ that represents a considerable burden of disease and still lacks adequate understanding and appropriate preventive or curative solutions. In addition to its direct health consequences, COVID-19 has disrupted economic activity, social interactions and political processes, affected civil liberties and interrupted education at all levels^22–26^. Although many governments and individuals no longer have the same level of concern as earlier in the pandemic^27^, many public health leaders, including members of this panel^28^, continue to regard COVID-19 as a persistent and dangerous health threat^29–31^.

Responses to the COVID-19 pandemic have been hindered by interrelated factors that include false information^32^, vaccine hesitancy^33,34^, inconsistent global coordination^35^, and the inequitable distribution of supplies^36^, vaccines^37,38^ and treatments^39^. Despite increased levels of trust in science during the pandemic^23,40^, there is information fatigue^4^ and waning compliance with those public health and social measures^41–43^ that remain in place, particularly those that affect daily lives^44^. Meanwhile, during periods of high community transmission, needs for services continue to exceed the capacity of many health systems^45^, which also are challenged by ongoing risks to the health of their workers^46–48^. Furthermore, long-standing social inequities have caused some populations to experience greater risk of COVID-19 infection, severe disease and death^37^. Many of these populations continue to have less access to COVID-19 vaccines^37,49^ and treatment^39^, as well as to resources to mitigate the mental health, social and economic consequences of the pandemic^50–52^.

Beneficial knowledge about COVID-19 aetiology, pathophysiology, prevention, vaccination, treatment and care has rapidly advanced through rigorous scientific, medical and public health inquiry, debate and collaboration^53–56^. Notwithstanding these advances, the responses of individual countries have been heterogeneous and often inadequate, in part because they lack coordination and clear goals.

**Fig. 1:**
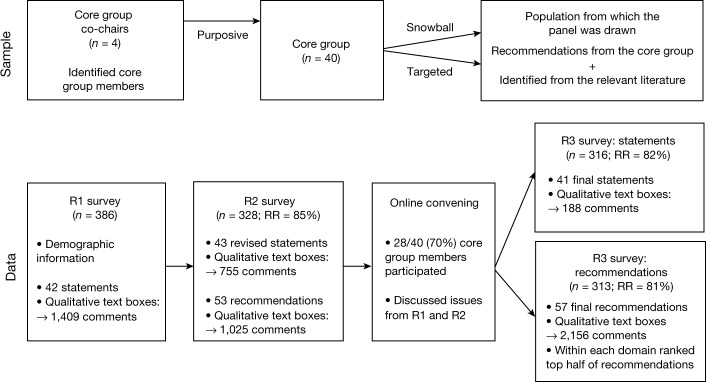
Delphi panel generation and data collection. Study methodology, including sample and data collection. Top, the iterative sampling approach used to generate a large, diverse Delphi panel (*n* = 386): four project co-chairs identified a core group of 40 academic, health, NGO, government and policy experts from 25 countries; the core group identified individuals with expertise in COVID-19; under-represented countries (that is, with fewer than one invitee) were identified and targeted through PubMed/Medline searches for authors of COVID-19 research studies in these countries. Bottom, the iterative digital data-collection process, including two survey rounds (R1 and R2) of draft statements; an online consensus meeting of the core group (Supplementary Discussion 3); one round of draft recommendations (R2); and a final survey round (R3) of the consensus statements and recommendations. Earlier rounds included text boxes for panellists to provide comments and suggest edits to individual statements (R1, R2) and recommendations (R2); the final statement and recommendations round (R3) allowed for overall comments at the end of each domain. For the final set of recommendations in R3, panellists ranked the top half in each of the six domains. RR, response rate.

To develop a global consensus regarding these ongoing problems, we carried out a Delphi study with a multidisciplinary, geographically diverse panel of 386 academic, health, non-governmental organization (NGO), government and other experts in COVID-19 response from 112 countries and territories (Table 1 and Methods). We achieved response rates of 85% in the second round (R2) and 82% and 81% in the third round (R3) surveys of the 41 statements and 57 recommendations, respectively. The mean levels of combined agreement (agree + somewhat agree) increased across the three rounds of the consensus statements (R1, 89%; R2, 90%; R3, 96%) and the two rounds of recommendations (R2, 93%; R3, 98%). The resulting consensus statements and recommendations (Fig. 1) can serve as a strong basis for decision-making to end COVID-19 as a public health threat, and permit a more durable resumption of social, cultural, religious, political, healthcare, economic and educational activities, with less burden on vulnerable populations.

**Table 1:** Expert panel characteristics (*n* = 386)

Characteristic	*n* (%)
**Gender**	
Man	225 (58)
Woman	155 (40)
No response	6 (2)
**Primary sector of employment**^a^	
Civil society	254 (66)
Private	61 (16)
Academic	39 (10)
Public	21 (5)
Other	6 (2)
No response	5 (1)
**Primary field**^b^	
Public health	156 (41)
Clinical research/care	92 (24)
Health policy/advocacy	67 (17)
Basic/physical/mathematical sciences	41 (11)
Other	24 (6)
No response	6 (2)
**Country income level**^c^	
Low- or middle-income country	195 (51)
High-income country	186 (48)
No response	5 (1)
**Global region of origin**^c^	
Europe and Central Asia	117 (30)
Latin America and Caribbean	56 (15)
East Asia and Pacific	49 (13)
North America	47 (12)
Sub-Saharan Africa	44 (11)
Middle East and North Africa	33 (9)
South Asia	35 (9)
No response	5 (1)

Percentages may not sum to 100 owing to rounding.

^a^Panellists were provided with these four standard categories for public health sectors and were able to provide a different response with the ‘other’ option.

^b^Panellists were provided with six response options (clinical research, public health research, healthcare provider, advocacy, health department or ministry and health policy) and ‘other’. The text responses under the ‘other’ option (*n* = 76) were analysed and recategorized into the four categories reported in the table.

^c^Country income level and global region correspond to World Bank classification for 2022 (https://datahelpdesk.worldbank.org/knowledgebase/articles/906519-world-bank-country-and-lending-groups).

## Top-ranked consensus recommendations

This multidisciplinary and multinational consensus study yielded 41 statements (Tables 2 and 3) and 57 forward-looking recommendations (Tables 4–7) on ending COVID-19 as a threat to public health grouped into six domains. Although we suggest that policymakers and other interested stakeholders review and consider the entire study findings, for expediency, we break out the top 10 recommendations ranked by the panellists in Table 8.

**Table 2:** Consensus statements to end COVID-19 as a public health threat

Statement		Grade	A (%)	SA (%)	SD (%)	D (%)	N (%)	NQ (%)
**Communication**								
STMT1.1	The volume and velocity of information during the COVID-19 pandemic have made it difficult for people to assess the accuracy of information.	A	81	19	0	1	316	0
STMT1.2	Public health authorities contribute to the dissemination of false information when their communications do not reflect current scientific understanding that transmission of SARS-CoV-2 is primarily airborne.	A	68	24	6	3	313	1
STMT1.3	Governments have inconsistently counteracted false information in the context of the COVID-19 pandemic.	A	70	23	4	3	312	1
STMT1.4	Sources of false information undermine the social cohesion needed for an effective public health response.	A	91	8	1	0*	316	0
STMT1.5	During the pandemic, public health officials have ineffectively engaged populations that have low levels of trust in government.	A	69	25	4	1	312	1
STMT1.6	Blaming unvaccinated individuals for continuation of the pandemic shifts attention away from government accountability.	B	57	31	8	4	307	3
STMT1.7	A government’s decision to reduce COVID-19 pandemic control measures does not mean that the threat to public health has ended.	A	94	5	1	0	315	0
**Health systems**								
STMT2.1	The world has not implemented an evidence-based, globally agreed-upon set of minimum COVID-19 pandemic response standards addressing monitoring, prevention, treatment and care.	A	73	18	6	3	309	1
STMT2.2	There continue to be systemic risks of COVID-19 infection for healthcare workers in many healthcare settings.	A	82	16	2	1	306	2
STMT2.3	Health systems are continuing to face abnormal staffing shortages due to the mental and physical health impacts on their workers from the COVID-19 pandemic.	A	79	16	4	0	305	3
STMT2.4	Healthcare workers continue to experience unaddressed mental health issues due to the pandemic.	A	81	17	2	0	296	5
STMT2.5	Governments have not always addressed the high out-of-pocket expenditure to consumers for some pandemic control measures (for example, testing) and personal protective equipment (for example, facemasks).	A	78	17	3	2	310	1
STMT2.6	The COVID-19 pandemic continues to reveal vulnerabilities in the global supply-chain framework for essential public health supplies.	A	91	8	1	0*	306	2
STMT2.7	The COVID-19 pandemic has catalysed opportunities for rapid innovation in digital health solutions throughout the care continuum.	A	84	14	1	1	308	1
STMT2.8	Leveraging economies of scale and scope through multicountry pooled procurement can enable health systems to increase access to essential medicines and supplies during public health crises.	A	85	14	1	1	305	2
STMT2.9	Community-based interventions and services to address the pandemic continue to be underused by health systems.	A	79	19	2	0*	302	4
**Vaccination**								
STMT3.1	When the risk of harm to others is sufficiently severe, governments may determine that the right of all individuals to good health overrides the autonomy of any one individual to choose not to be vaccinated.	A	68	24	5	4	309	1
STMT3.2	Individual medical autonomy acknowledges that individuals who have decision-making capacity have the right to make decisions regarding vaccination, even when their decisions contradict their healthcare providers’ recommendations.	B	59	25	8	8	306	2
STMT3.3	Vaccine hesitancy, which ranges from delay to refusal despite the availability of vaccine services, remains a major challenge to ending the COVID-19 pandemic as a public health threat.	A	75	21	3	1	309	1
STMT3.4	Discussing vaccine hesitancy as primarily a function of information or worldview is inaccurate, as vaccine hesitancy is a multifactorial phenomenon comprising other factors (for example, socioeconomic).	A	82	12	3	2	308	1
STMT3.5	Continued low levels of trust in information from government sources are associated with vaccine hesitancy.	A	78	17	4	2	309	1
STMT3.6	Vaccination alone is insufficient to end the COVID-19 pandemic as a public health threat.	A	83	14	3	1	311	0

Grades are based on the percentage of combined agreement (agree + somewhat agree). U, unanimous (100%) agreement; A, 90%–99% agreement; B, 78%–89% agreement; C, 67%–77% agreement. Responses to each statement (STMT) are presented as percentages of the total responses. A, agree; SA, somewhat agree; SD, somewhat disagree; D, disagree; N, total number of responses; NQ, the number of participants that indicated that they were not qualified to respond. The asterisks indicate that rounding resulted in 0% despite the presence of ≥1 response in the disagreement category.

**Table 3:** Consensus statements to end COVID-19 as a public health threat

Statement		Grade	A (%)	SA (%)	SD (%)	D (%)	N (%)	NQ (%)
**Prevention**								
STMT4.1	SARS-CoV-2 is an airborne virus that presents the highest risk of transmission in indoor areas with poor ventilation.	A	92	8	0*	0*	311	0
STMT4.2	The assumption that endemicity automatically means that variants will have lower virulence is not scientifically sound and should not be a basis for public policy decision-making.	A	81	15	2	1	297	5
STMT4.3	SARS-CoV-2 mammal-to-mammal, outdoor transmission represents a reservoir for future zoonotic variants.	A	76	20	3	1	268	14
STMT4.4	Relying on individual, voluntary compliance with transmission prevention measures is insufficient to end COVID-19 as a public health threat.	A	81	15	3	1	311	0
STMT4.5	Infection rates tend to increase when governments discontinue social measures, including non-pharmaceutical interventions, regardless of the level of vaccination.	A	75	19	4	2	306	2
STMT4.6	Wide use of high-filtration and well-fitting facemasks (for example, N95, KF94, KN95, FFP2/3) is important to reduce transmission, particularly in high-risk settings.	A	87	9	3	1	307	1
STMT4.7	Most countries have not adequately protected children throughout the pandemic, that is, preventing SARS-CoV-2 transmission while simultaneously addressing their physical, mental and social well-being.	A	77	17	5	2	309	1
**Treatment and care**								
STMT5.1	Prioritizing the treatment of severe COVID-19 over the prevention of SARS-CoV-2 transmission risks increasing infections, long COVID and the overall burden of disease.	A	72	20	5	2	299	4
STMT5.2	More effective COVID-19 therapeutic options, as well as care delivery models, are needed.	A	91	8	1	0	303	3
STMT5.3	In addition to the standardized long COVID case definition for adults, a standardized definition is needed for children.	A	90	10	0*	0	298	4
STMT5.4	Research is needed to determine whether infection from distinct variants of SARS-CoV-2 is associated with significant differences in long-term morbidity.	A	91	8	1	0	305	2
**Pandemic inequities**								
STMT6.1	The COVID-19 pandemic disproportionately impacts the most vulnerable populations within communities, countries and globally.	A	92	6	1	1	311	0
STMT6.2	The decision by most high-income countries to protect intellectual property rights for COVID-19 vaccines and treatments has contributed to limited options available to low- and middle-income countries for addressing the pandemic.	A	83	11	4	3	304	3
STMT6.3	It is in the best interests of high-income countries to fund the equitable distribution of vaccines and treatments to low- and middle-income countries.	A	88	9	2	0*	308	1
STMT6.4	There is a disproportionate consumption of health system resources by those voluntarily unvaccinated.	A	65	25	7	3	295	5
STMT6.5	When expanding use of digital communications technology (for example, online appointment systems, mobile patient communications and telehealth applications) health systems may inadvertently contribute to inequitable access to healthcare services.	A	64	30	6	1	307	1
STMT6.6	The global pandemic response has generally not taken into account the underlying role of social determinants of health.	A	77	21	2	0*	308	1
STMT6.7	Few governments have adequately engaged vulnerable populations to inform pandemic response priorities.	A	78	17	4	0*	303	3
STMT6.8	The incorporation of research paradigms from diverse disciplines has greater potential to end COVID-19 as a public health threat than reliance on a single research paradigm (for example, evidence-based medicine).	A	88	9	2	1	309	1

Grades are based on the percentage of combined agreement (agree + somewhat agree). U, unanimous (100%) agreement; A, 90%–99% agreement; B, 78%–89% agreement; C, 67%–77% agreement. Responses to each statement (STMT) are presented as percentages of the total responses. A, agree; SA, somewhat agree; SD, somewhat disagree; D, disagree; N, total number of responses; NQ, the number of participants that indicated that they were not qualified to respond. The asterisks indicate that rounding resulted in 0% despite the presence of ≥1 response in the disagreement category.

**Table 4:** Recommendations to end COVID-19 as a public health threat

Recommendation		Grade	A (%)	SA (%)	SD (%)	D (%)	N (%)	NQ (%)	Rank
**Communication**									
REC1.1	Community leaders, scientific experts and public health authorities should collaborate to develop public health messages that build and enhance individual and community trust and use the preferred means of access and communication for different populations.	U	96	4	0	0	312	0	1
REC1.2	Public health authorities should partner with individuals and organizations that are trusted within their communities to provide accurate, accessible information about the pandemic and inform behaviour change.	A	95	5	0*	0	312	0	2
REC1.3	Public health professionals and authorities should combat false information proactively based on clear, direct, culturally responsive messaging that is free of unnecessary scientific jargon.	A	94	5	0*	0*	311	0	3
REC1.4	Institutions and individuals that wish to advance public trust should: (1) draw on evidence about how trust is created and restored; (2) provide training and professional development emphasizing skills and competencies that convey trustworthiness; and (3) develop, implement and assess communication strategies that are highly likely to create or restore trust.	A	94	5	1	0	311	0	4
REC1.5	Multidisciplinary researchers should assess the impact of the ‘infodemic’ on health behaviours and outcomes in specific populations of all countries.	A	93	6	1	0*	311	0	
REC1.6	Research funders should commission more scoping, narrative and systematic reviews to synthesize, evaluate and disseminate COVID-19-related evidence.	A	83	16	1	0*	309	0	
REC1.7	Governments should determine which agencies are or should be accountable for monitoring health information and develop monitoring tools to identify false information.	A	81	17	2	1	312	1	5
REC1.8	Social media companies should engage transparently with researchers and developers, who are free of a direct conflict of interest, to implement controls for their platforms that reduce publication and dissemination of false health information.	A	87	11	2	1	311	0	
REC1.9	Governments, industry and non-governmental organizations should actively identify and expose individuals and networks that promote false health information about the COVID-19 pandemic.	A	80	17	2	1	310	1	
REC1.10	Governments should consider holding publishers of false health information liable, while balancing civil liberties.	A	76	17	6	1	308	1	
**Health systems**									
REC2.1	Governments should remove economic barriers to SARS-CoV-2 tests, personal protective equipment, treatments and care.	A	90	10	0*	0	313	0	6
REC2.2	Governments and global health organizations should support the development of regional hubs for the manufacturing of COVID-19 supplies, treatments and vaccines.	A	91	8	0	0*	308	2	5
REC2.3	The user experience and interface with digital health technologies should be adapted to expand access for all, with particular attention to vulnerable groups.	A	86	13	0*	0*	313	0	
REC2.4	Healthcare organizations should support their workers’ physical, mental and social well-being.	A	97	2	0	1	311	0	4
REC2.5	Pandemic preparedness and response planning should adopt a whole-of-society approach that includes multiple disciplines, sectors and actors (for example, business, civil society, engineering, faith communities, mathematical modelling, military, media and psychology).	A	95	4	1	0	312	0	1
REC2.6	Preparedness and response strategies should adopt whole-of-government approaches (for example, multiministry coordination) to identify, review and address resilience in health systems.	A	93	6	1	0	309	2	2

Grades are based on the percentage of combined agreement (agree + somewhat agree). U, unanimous (100%) agreement; A, 90%–99% agreement; B, 78%–89% agreement; C, 67%–77% agreement. Responses to each recommendation (REC) are presented as percentages of the total responses. A, agree; SA, somewhat agree; SD, somewhat disagree; D, disagree; N, total number of responses; NQ, the number of participants that indicated that they were not qualified to respond. The asterisks indicate that rounding resulted in 0% despite the presence of ≥1 response in the disagreement category.

**Table 5:** Recommendations to end COVID-19 as a public health threat

Recommendation		Grade	A (%)	SA (%)	SD (%)	D (%)	N (%)	NQ (%)	Rank
**Health systems**									
REC2.7	As social, political and economic sector risks continue to have spillover effects on health systems, key multisector indicators for systemic risks to health systems must be identified and assessed.	A	92	7	1	0*	305	2	
REC2.8	The identification of several variants of concern necessitates substantial virological surveillance based on whole-genome sequencing of positive specimens.	A	86	13	1	0	287	8	
REC2.9	Public health policy should take better account of the potential long-term impact of the unchecked spread of COVID-19, given ongoing uncertainties about the prevalence, severity and duration of post-COVID-19 morbidity (long COVID).	A	86	13	1	0*	310	1	
REC2.10	To reduce the burden on hospitals, primary care should be strengthened to include testing, contact tracing, the monitoring of mild symptoms and vaccination.	A	92	6	1	0*	310	1	3
REC2.11	Governments and industry should engage continuous improvement disciplines for intercountry procurement, pooling and supply chain management to reduce cycle times and costs, as well as improve product quality and data to rapidly scale up the availability of medicines, protective equipment and vaccines.	A	91	7	2	0	301	4	8
REC2.12	Public health systems should prioritize the use of implementation science to determine which digital health solutions can and should be quickly scaled up globally.	A	85	13	2	0	300	4	
REC2.13	Investments in digital health infrastructure, software and training should be made to institutionalize quality telehealth and telemedicine services.	A	86	12	2	0	310	1	
REC2.14	To reduce the burden on health systems and healthcare workers, community-based organizations and students pursuing degrees in health-related fields should be engaged to educate, test and vaccinate the population.	A	77	20	3	0*	312	0	
REC2.15	Health systems should identify and, where possible, reduce diagnostic, treatment and care backlogs for non-COVID-19-related medical conditions.	A	88	9	1	3	305	3	
REC2.16	Because the global marketplace has not satisfied demand for vaccines, treatments and supplies, countries and regions should consider legislative and regulatory reforms to address these market failures (for example, nationalizing manufacturing capacity, negotiating global and regional trade agreements, adjusting intracountry intellectual property rights).	A	80	13	4	3	297	6	7
REC2.17	In the absence of a new multilateral organization focused on pandemic control, Member States should authorize the WHO to lead a large, inclusive, multistakeholder, global effort to provide public health and clinical targets pertaining to the pandemic, with an emphasis on cases, vaccination, morbidity and mortality.	A	73	19	6	2	306	3	9
REC2.18	In settings in which access to PCR or antigen tests may be limited, providers should consider adopting a syndromic approach to COVID-19 diagnosis for symptomatic individuals.	B	68	21	9	2	282	11	

Grades are based on the percentage of combined agreement (agree + somewhat agree). U, unanimous (100%) agreement; A, 90%–99% agreement; B, 78%–89% agreement; C, 67%–77% agreement. Responses to each recommendation (REC) are presented as percentages of the total responses. A, agree; SA, somewhat agree; SD, somewhat disagree; D, disagree; N, total number of responses; NQ, the number of participants that indicated that they were not qualified to respond. The asterisks indicate that rounding resulted in 0% despite the presence of ≥1 response in the disagreement category.

**Table 6:** Recommendations to end COVID-19 as a public health threat

Recommendation		Grade	A (%)	SA (%)	SD (%)	D (%)	N (%)	NQ (%)	Rank
**Vaccination**									
REC3.1	Vaccination messaging should clearly explain the efficacy and limitations of current vaccines in preventing SARS-CoV-2 transmission and reducing the severity of COVID-19.	A	93	7	0*	0*	312	0	2
REC3.2	In settings where individuals have lower levels of trust in government, vaccination efforts should engage trusted local leaders and organizations.	A	93	6	1	0	311	0	
REC3.3	To combat vaccine hesitancy, tailored messages that address the underlying bases of an individual’s concerns should be used in targeted public health communications.	A	93	6	1	0	310	1	3
REC3.4	Government, philanthropic and industry funding should include a focus on developing vaccines that provide long-lasting protection against multiple SARS-CoV-2 variants.	A	90	9	1	0	309	1	1
REC3.5	Calculations for immunity should take into consideration the time following the date of vaccination and/or infection and be regularly updated with new scientific evidence.	A	93	4	2	1	398	4	
REC3.6	As the causes of vaccine hesitancy are not solely a function of information or worldview, economic incentives should be considered in parallel with information and access to increase vaccination rates.	B	57	25	13	5	303	3	
**Prevention**									
REC4.1	Governments should regulate and incentivize the development and deployment of structural prevention measures (for example, ventilation, air filtration) to mitigate airborne transmission of SARS-CoV-2, with an early emphasis on high-risk settings.	A	86	12	1	0	307	0	
REC4.2	Measures that are no longer scientifically valid for COVID-19 prevention should be immediately removed from COVID-19 guidance and policy.	A	88	10	2	0	307	0	
REC4.3	Risk communications should clearly emphasize that transmission of SARS-CoV-2 is primarily caused by inhalation of the virus.	A	90	8	2	0	302	1	
REC4.4	National and international travel restrictions should be based on current scientific knowledge and prevailing transmission rates of all variants that take into account relevant, health-based factors (for example, traveller’s vaccination status, proof of recent recuperation from COVID-19 or a negative result of an antigen or PCR test).	A	85	12	1	2	305	0	
REC4.5	All countries should adopt a vaccines-plus approach that includes a combination of COVID-19 vaccination, prevention measures, treatment and financial incentives.	A	82	14	4	0	307	0	1
REC4.6	Prevention of SARS-CoV-2 transmission in the workplace, educational institutions and centres of commerce should remain a high priority, reflected in public health guidance and supported through multiple social measures and structural interventions (for example, remote work/schooling policies, ventilation, air filtration, facemask wearing).	A	85	11	3	1	307	0	2
REC4.7	Governments should consider imposing broad restrictions on civil liberties only in the event of variants of concern presenting risk of high rates of transmission and severity, coupled with (1) waning immunity or (2) vaccine resistance.	A	71	21	5	3	305	0	3

Grades are based on the percentage of combined agreement (agree + somewhat agree). U, unanimous (100%) agreement; A, 90%–99% agreement; B, 78%–89% agreement; C, 67%–77% agreement. Responses to each recommendation (REC) are presented as percentages of the total responses. A, agree; SA, somewhat agree; SD, somewhat disagree; D, disagree; N, total number of responses; NQ, the number of participants that indicated that they were not qualified to respond. The asterisks indicate that rounding resulted in 0% despite the presence of ≥1 response in the disagreement category.

**Table 7:** Recommendations to end COVID-19 as a public health threat

Recommendation		Grade	A (%)	SA (%)	SD (%)	D (%)	N (%)	NQ (%)	Rank
**Treatment and care**									
REC5.1	Global case definitions for SARS-CoV-2 and for COVID-19 morbidity and mortality should be standardized.	U	92	8	0	0	305	0	3
REC5.2	Promote multisectoral collaboration to accelerate the development of new therapies for all stages of COVID-19 (for example, outpatient, hospitalization and long COVID).	A	95	5	0	0*	309	1	1
REC5.3	Clinical trials and longitudinal cohorts should include statistically sufficient samples from all age groups, genders and vulnerable populations.	A	93	7	0*	0	306	1	
REC5.4	Expand the evidence base on the cumulative effect of COVID-19 reinfection to inform public health policy.	A	90	9	1	0*	308	1	
REC5.5	Governments should now prioritize early case detection so that health systems can facilitate earlier treatment and care.	A	80	17	1	1	304	1	
REC5.6	Prioritize research funding for long COVID to develop diagnostic tools, treatment and care, and knowledge about extrinsic factors (for example, stigma and discrimination).	A	85	12	3	0	306	0	2
**Pandemic inequities**									
REC6.1	Recognizing that local and regional contexts are important for equitable responses to the pandemic, governments should engage communities and multidisciplinary experts who understand the local context when developing operational plans for ending COVID-19 as a public health threat.	A	95	5	0*	0	311	0	3
REC6.2	In addition to current vaccine equity efforts, governments and global health organizations should better coordinate to make COVID-19 tests and treatments affordable for all people in all countries.	A	93	6	0*	0	310	0	4
REC6.3	Decision-making bodies (for example, governments, WHO committees) should meaningfully and transparently engage with a broad base of voices to inform their decisions.	A	93	6	0*	0	311	0	
REC6.4	Governments, regional bodies, industry and health systems should anticipate the procurement and supply management needs for supplies, treatments and vaccines in low-resource settings (for example, transportation logistics, storage, refrigeration).	A	93	6	0*	0*	306	2	
REC6.5	Pandemic preparedness, response planning and policy should be reviewed and updated to protect children, emphasizing the prevention of SARS-CoV-2 transmission while simultaneously addressing their physical, mental and social well-being.	A	90	9	0*	1	309	0	
REC6.6	Global trade and health organizations should coordinate with countries to negotiate the transfer of technologies enabling manufacturers in low- and middle-income countries to develop quality assured and affordable vaccines, tests and therapeutics.	A	95	4	1	0*	307	2	2
REC6.7	Pandemic preparedness and response should address pre-existing social and health inequities.	A	94	5	1	0*	307	1	1
REC6.8	Governments, industry and health systems should prioritize minimizing closed- and open-vial vaccine wastage, with an early emphasis on wastage resulting from unnecessarily short expiration dates, and by addressing regulatory barriers and procurement and supply management challenges for transferring or donating vaccine doses.	A	86	13	1	0	301	3	
REC6.9	Pandemic preparedness and response efforts should assess and mitigate the risks and effects of SARS-CoV-2 transmission among people within and emigrating from conflict zones.	A	86	13	2	0		1	
REC6.10	High-income countries should refocus the distribution of vaccines to countries with low rates of vaccination and inadequate access to vaccines.	A	86	12	2	0*		0	5

Grades are based on the percentage of combined agreement (agree + somewhat agree). U, unanimous (100%) agreement; A, 90%–99% agreement; B, 78%–89% agreement; C, 67%–77% agreement. Responses to each recommendation (REC) are presented as percentages of the total responses. A, agree; SA, somewhat agree; SD, somewhat disagree; D, disagree; N, total number of responses; NQ, the number of participants that indicated that they were not qualified to respond. The asterisks indicate that rounding resulted in 0% despite the presence of ≥1 response in the disagreement category.

The top three recommendations focus on whole-of-society^1^ action and maintaining, or in some cases returning, to a vaccines-plus approach^2^. First, to avoid the inefficiency and ineffectiveness of fragmented efforts, pandemic preparedness and response should adopt a whole-of-society strategy that includes multiple disciplines, sectors and actors. Second, going forward, whole-of-government approaches (such as interministry coordination) can identify, review and address resilience in health systems to make them more responsive to people’s needs. Third, all countries should adopt a vaccines-plus approach, which includes a combination of COVID-19 vaccination, other prevention measures, treatment and financial incentives such as support measures. Infection rates tend to increase when governments discontinue social measures, including non-pharmaceutical interventions, regardless of the level of vaccination^57,58^.

**Extended Data Fig. 1:**
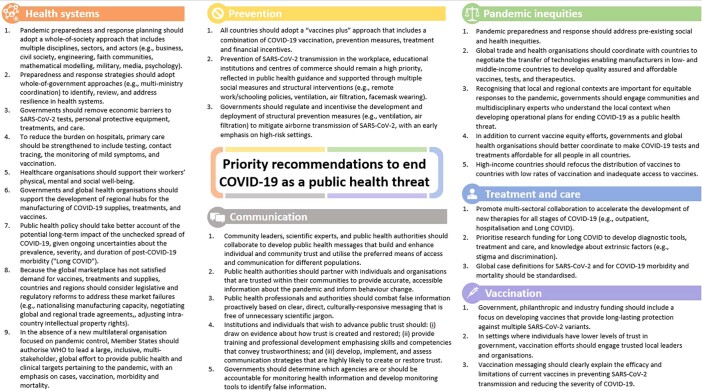
Top half of the ranking of the recommendations in each domain. In the third and final round of the Delphi process, panel members were asked to rank the recommendations per domain (n = 6) based on importance. This figure shows the top half of the recommendations for each of the six domains (communication; health systems; vaccination; prevention; treatment and care; and inequities).

The degree of consensus achieved for statements and recommendations, along with a ranking exercise in the final round, informed our synthesis of the study’s findings into six cross-cutting themes (Box 1) to which we believe decision-makers should pay particular attention: (1) SARS-CoV-2 is still present among us—despite some governments moving on—requiring continued efforts and resources to save lives; (2) vaccines are an effective tool against COVID-19 but will not alone end COVID-19 as a public health threat; (3) multisectoral collaboration that centres on communities and fosters trust is needed; (4) responsive health systems are crucial for responding to the COVID-19 pandemic and require coordinated government support; (5) adverse forces challenge efforts to end the COVID-19 public health threat; and (6) none of us is safe until everyone is safe. For ease of review, we report the tophalf ranked recommendations within each domain (Extended Data Fig. 1).

Box 1  Cross-cutting themes for action to end COVID-19 as a public health threat

1. SARS-CoV-2 still moves among us—despite some governments moving on—requiring continued efforts and resources to save lives. Reservoirs exist from which variants of concern may yet emerge^104,105^; possible endemicity^45^ does not necessarily mean lower disease severity^106^. Broad-based funding to develop long-lasting immunogenic vaccines must proceed concurrent with other prevention measures. The long-term impact of infection must be assessed, as long COVID has emerged as a chronic condition^107–110^.
2. Vaccines are an effective tool against COVID-19 but will not alone end COVID-19 as a public health threat. Vaccination as a sole pandemic response strategy has limitations due to immune escape^111–113^, waning immunity^17,114,115^, inequitable access^34,116^, vaccine hesitancy^117–120^ and the absence of immunization strategies^121^. A multifaceted public health vaccines-plus approach is needed, including testing, surveillance, treatment^122^, community engagement and implementation of social prevention measures (such as facemasks^123,124^, distancing and quarantine), structural interventions (such as ventilation and air filtration)^2^ and financial incentives (for example, support measures).
3. Multisectoral collaboration that centres on communities and fosters trust is needed. Ending COVID-19 as a public health threat requires whole-of-society and whole-of-government approaches engaging trusted community leaders and organizations, scientific experts, businesses, and other disciplines and sectors^1,125^. This expanded pool of collaborators can best address diverse needs regarding modes of access, communication, innovation and trust among different populations^126,127^.
4. Responsive health systems are crucial for responding to the COVID-19 pandemic and require coordinated government support. The persistent demand on health systems requires protecting the physical and mental wellbeing of healthcare workers; reducing economic barriers for equipment and treatment, including addressing supply-chain factors^128^; strengthening primary care; and adopting a comprehensive, intersectoral, multilevel approach to preparedness and response activities.
5. Adverse forces challenge efforts to end the COVID-19 public health threat. Counteract sovereign state actors who are openly antagonistic toward science and public health and other entities with vested interests that disseminate false information. Public health authorities should build trust in evidence-based communications and partner with those monitoring and holding accountable disseminators of false information^129^.
6. None of us is safe until everyone is safe. Pandemic inequities must end. This includes taking into account pre-existing social determinants of health, addressing access to affordable vaccines, tests, other supplies and treatment^50,130^, and paying special attention to the needs of vulnerable groups (such as older^131,132^ and immunocompromized^133^ individuals, children^134^ and healthcare workers^48,135,136^).

## Areas of less agreement

The Delphi process involves a review and revision methodology that can result in relatively greater agreement among statements and recommendations over successive survey rounds while also identifying areas of disagreement that may require special efforts going forward. In addition to its the four-point Likert agreement–disagreement response options available in this study, panellists could select ‘not qualified to respond’ for items that they perceived as falling outside their expertise (see the ‘Delphi expert panel member sample’ section in the Methods). Although our study reflects relatively few areas of disagreement, we believe that highlighting the key areas of disagreement may be instructive for decision-makers in their own prioritization processes addressing the COVID-19 pandemic.

Extended Data Table 1 presents the six recommendations reflecting 5% or greater disagreement (disagree + somewhat disagree). Of those six, only two recommendations had greater than 10% disagreement: 18% of panellists disagreed with the recommendation to consider further economic incentives to potentially address vaccine hesitancy (REC3.6) and 11% disagreed with the recommendation that providers adopt a syndromic approach to COVID-19 diagnosis in settings with lower access to testing (REC2.18). The remaining four recommendations broadly relate to the use of governmental regulatory and enforcement powers in disease control efforts.

For statements and recommendations with response rates of ‘agree’ alone (that is, not combined with ‘somewhat agree’) below 67%, we conducted bivariate analyses to examine potential associations with panellist demographics; six statements (STMT1.2, STMT1.3, STMT2.1, STMT2.3, STMT3.5, STMT6.6) and one recommendation (REC4.5) demonstrated significant differences. Respondents who disagreed were significantly more likely to work in low- and middle-income countries than in high-income countries (*P* < 0.05; Supplementary Discussion 2). Few differences in agreement were identified by sector or field of employment, except for STMT1.1, for which greater disagreement was identified among those working in the health policy/advocacy field, and for STMT1.3, for which the academic and public sectors evidenced greater disagreement than other sectors.

## Key statements and recommendations

The following six domains summarize the main areas of agreement, with a particular focus on the recommendations. The quantitative results on agreement and disagreement for the statements and recommendations are reflected in the tables and are further illustrated in Supplementary Discussion 1.

### Communicate effectively

Substantial combined agreement among the panellists (range, 88–100%) indicates that communication issues remain a key area of risk and opportunity for ending COVID-19 as a public health threat. Policymakers and public health agencies should take special care when communicating the causation of and continuing accountability for the pandemic (Tables 2 (STMT1.7) and 4 (REC1.1)). The lowest level of agreement in this domain (agree, 57%; combined agreement, 88%) was found for a statement about government accountability receiving less attention when unvaccinated individuals are blamed for the pandemic’s continuation (Table 2 (STMT1.6)).

The panel focused primarily on the role of trust in government (Table 2 (STMT1.5)), the consequences of false information (Table 2 (STMT1.2, STMT1.3, STMT1.4)) and the rapid production of large volumes of new COVID-19-related information (Table 2 (STMT1.1)). That said, governments themselves may be a source of misinformation, for example, in the context of identifying transmission mechanisms (Table 6 (REC4.3)) and when stating that the COVID-19 pandemic has ended (Table 2 (STMT1.7)).

To counteract the infodemic and false information, governments should monitor false information (Table 4 (REC1.7)), expose networks of false information (Table 4 (REC1.9)) and consider holding publishers of false information liable (Table 4 (REC1.10)). Furthermore, public health professionals and other authorities should use clear, culturally responsive messaging to combat false information (Table 4 (REC1.3)). In parallel, social media companies should implement controls that reduce the publication and dissemination of false health information (Table 4 (REC1.8)).

Institutions and individuals should advance public trust by seeking training on building trust and developing trust-oriented communication strategies (Table 4 (REC1.4)), expanding collaboration with community leaders and the scientific community (Table 4 (REC1.1)), and working with individuals and organizations that have established trust in communities (Table 4 (REC1.2)). Using the preferred means of communication for different populations was unanimously recommended to further earn trust (Table 4 (REC1.1)).

Multidisciplinary research should assess the impact of the COVID-19 infodemic on health behaviours and outcomes (Table 4 (REC1.5)). Research funders should commission more reviews that synthesize, evaluate and disseminate COVID-19-related evidence to inform needed interventions (Table 4 (REC1.6)).

### Strengthen health systems

Health systems have experienced wide-ranging circumstances throughout the pandemic, from periods of relative calm to periods of near collapse. The broad agreement among panellists strongly suggests that, although many health systems will remain at risk of once again being overwhelmed, those risks can be mitigated. Certain sources of risk to health systems are essentially structural, such as the lack of implementation of an evidence-based, globally agreed-upon set of minimum COVID-19 pandemic response standards (Table 2 (STMT2.1)).

As noted above, health systems recommendations with respect to whole-of-society (Table 4 (REC2.5)) and whole-of-government approaches (for example, multiministry coordination) (Table 4 (REC2.6)) were among the most highly ranked by the panel.

As community transmission of SARS-CoV-2 continues to present a risk to health systems, particularly through variants of concern, extensive virological surveillance should be used (Table 5 (REC2.8)). Public health policies should take better account of the potential long-term impact of the unchecked spread of COVID-19 given the ongoing uncertainties about the prevalence, severity and duration of post-COVID-19 morbidity (long COVID) (Table 5 (REC2.9)). Member States should authorize the World Health Organization (WHO) to lead a large, inclusive, multistakeholder, global effort to provide public health and clinical targets pertaining to SARS-CoV-2 and COVID-19, with an emphasis on cases, vaccination, morbidity and mortality (Table 5 (REC2.17)).

Economic impacts, notably costs borne by consumers (Table 2 (STMT2.5)), create risks to health systems. To address these risks, structural and economic recommendations include removing economic barriers to SARS-CoV-2 tests, personal protective equipment, treatment and care (Table 4 (REC2.1)), supporting the development of regional manufacturing hubs for COVID-19 supplies, treatments and vaccines (Table 4 (REC2.2)), and considering legislative and regulatory reforms to address market failures (Table 5 (REC2.16)). Where access to PCR or antigen tests is limited, providers should consider adopting a syndromic approach (Table 5 (REC2.18)). Notably, REC2.18 is the health systems recommendation with the highest percentages of panellists disagreeing as well as panellists indicating ‘not qualified to respond’.

To reduce the burden on hospitals, the role of primary health care should be strengthened (Table 5 (REC2.10)), while health care workers’ physical, mental and social well-being should be supported (Table 4 (REC2.4)).

With respect to digital health, the recommendations encourage increasing investments in digital health infrastructure (Table 5 (REC2.13)), adapting user interfaces and experience to expand access, particularly for vulnerable groups (Table 4 (REC2.3)), and leveraging implementation science to determine which digital health solutions can be quickly scaled (Table 5 (REC2.12)).

With respect to procurement practices, engaging continuous improvement disciplines for intercountry procurement, pooling and supply-chain management was urged (Table 5 (REC2.11)). To best leverage community-based interventions and services, community-based organizations and students pursuing degrees in health-related fields should be engaged in providing COVID-19 education, testing and vaccination services (Table 5 (REC2.14)).

As social, political and economic sector risks continue to have spillover effects on health systems, key multisectoral indicators for systemic risks to health systems should be identified and assessed (Table 5 (REC2.7)).

Finally, health systems should identify and, where possible, reduce diagnostic, treatment and care backlogs for non-COVID-19-related medical conditions (Table 5 (REC2.15)).

### Emphasize vaccination, but not exclusively so

Even assuming continued innovation of vaccines and interventions that reduce vaccine hesitancy, 97% of the panel agrees that vaccination alone is insufficient to end the COVID-19 pandemic as a public health threat (Table 2 (STMT3.6)). Thus, the panel places a strong emphasis on additional prevention measures, particularly, as noted above and in the ten highest-ranked recommendations (Table 8), for countries to adopt a vaccines-plus approach, as discussed in the next domain.

Regarding the key role of vaccines, the panel made a range of recommendations. Government, philanthropic and industry funding should invest in developing vaccines that provide long-lasting protection against multiple SARS-CoV-2 variants (Table 6 (REC3.4)). As waning immunity remains a risk, calculations for immunity should consider the time after the date of vaccination and/or infection and be regularly updated with new scientific evidence (Table 6 (REC3.5)).

Vaccine hesitancy, which ranges from delay to refusal despite availability of vaccine services, remains a major challenge (Table 2 (STMT3.3)). To reduce vaccine hesitancy and increase uptake, several interventions are recommended: engaging trusted local leaders and organizations in vaccination efforts (Table 6 (REC3.2)), providing information that clearly explains the efficacy and limitations of current vaccines (Table 6 (REC3.1)) and tailoring messages to address the underlying bases of various populations’ specific concerns through targeted public health communications (Table 6 (REC3.3)). Vaccine hesitancy may also be associated with false information, which is addressed in the communication domain above.

On the one hand, panellists largely agree that medical autonomy of individuals with decision-making ability extends to the right to make one’s own decisions regarding vaccination (Table 2 (STMT3.2)). On the other hand, panellists also acknowledge that, when the risk of harm to others is sufficiently severe, governments may determine that the right of all individuals to good health overrides the autonomy of any one individual to choose not to be vaccinated (Table 2 (STMT3.1)). These statements reflect among the highest levels of combined disagreement (Table 2 (STMT3.1, 9%; STMT3.2, 16%)). Civil liberties implications are further discussed in the next domain.

### Promote preventive behaviours

As noted above, vaccination alone will not end COVID-19 as a public health threat (Table 2 (STMT3.6)) for all people. Infection rates tend to increase when governments discontinue social measures, including non-pharmaceutical interventions, regardless of the level of vaccination (Table 3 (STMT4.5)). Thus, all countries should adopt a vaccines-plus approach, including a combination of COVID-19 vaccination, other prevention measures, treatment and possibly financial incentives (Table 6 (REC4.5)).

Although the nature and vectors of SARS-CoV-2 transmission were not clearly understood early in the pandemic, current evidence guided the panellists to near-unanimous agreement that SARS-CoV-2 is an airborne virus that presents the highest risk of transmission in indoor areas with poor ventilation (Table 3 (STMT4.1)). Risk-related communications from all actors should clearly emphasize that transmission of SARS-CoV-2 is primarily caused by inhalation of the virus (Table 6 (REC4.3)). Considering the airborne nature of transmission, governments should regulate and incentivise structural prevention measures, such as ventilation and air filtration (Table 6 (REC4.1)), and high priority should be given to preventing SARS-CoV-2 transmission in the workplace, educational institutions and commercial centres (Table 6 (REC4.6)).

Mammal-to-mammal transmission represents a reservoir for future zoonotic variants (Table 3 (STMT4.3)). Thus, substantial virological surveillance based on whole-genome sequencing of positive samples in human and high-risk mammal populations is an essential component of the continued pandemic response and preparedness (Table 5 (REC2.8)).

National and international travel restrictions should be based on current scientific knowledge and prevailing transmission rates of all variants that consider relevant, health-based factors (Table 6 (REC4.4)). Measures that are no longer scientifically valid for COVID-19 prevention should be immediately removed from COVID-19 guidance and policy (Table 6 (REC4.2)). Going forward, governments should consider imposing broad restrictions on civil liberties only in the event of variants of concern presenting risk of high rates of transmission and severity, coupled with waning immunity or vaccine resistance (Table 6 (REC4.7)).

### Expand treatments

Panellists had substantially high agreement regarding all aspects of treatment and care, indicating that treatment will continue to be an area of major importance both for ending COVID-19 as a public health threat and for individual patient care. Notably, a statement addressing the risk of prioritizing treatment over prevention (Table 3 (STMT5.1)) had the highest level of combined disagreement (7%) for this domain.

With current public health policies reflecting greater tolerance for community transmission and increased rates of infection, research into COVID-19 must adapt and develop further evidence to understand the cumulative effect of COVID reinfection (Table 7 (REC5.4)). Research is needed to determine whether infection from distinct variants of SARS-CoV-2 is associated with significant differences in long-term morbidity (Table 3 (STMT5.4)). Additional research funding, particularly for long COVID, should be prioritized (Table 7 (REC5.6)), and multisectoral collaboration should accelerate new therapies across all stages of COVID-19 (Table 7 (REC5.2)). Moreover, global case definitions should be standardized (Table 7 (REC5.1)).

Echoing some statements and recommendations in the pandemic inequities domain (discussed below), clinical trials and longitudinal cohorts should be more inclusive and statistically representative regarding age, gender and vulnerable populations (Table 7 (REC5.3)).

### Eliminate inequities

The substantial agreement of the panellists suggests that addressing inequities remains a global challenge. Immediate efforts should be made to reduce vaccine wastage (Table 7 (REC6.8)), addressing the need for cold storage, transport and other infrastructure-based barriers in low-resource settings (Table 7 (REC6.4)), addressing the affordability of testing and treatment for people in all countries (Table 7 (REC6.2)), as well as accelerating efforts to distribute vaccines in low- and middle-income countries (Table 7 (REC6.10)).

Transfer agreements to increase production capacities in low- and middle-income countries should be expedited (Table 7 (REC6.6)). Pre-existing social and health inequities must be considered in pandemic preparedness and response going forward (Table 7 (REC6.7)). The findings call special attention to two vulnerable populations: children (Table 7 (REC6.5)) and those living within or fleeing from conflict zones (Table 7 (REC6.9)).

The pandemic has illustrated the risk of over-reliance on experts from a small number of disciplines (Table 3 (STMT6.8)), often excluding the expertise of community members (Table  4 (REC1.2)) and vulnerable groups (Table 3 (STMT6.7)). Instead, vulnerable groups should be sought out and actively engaged (Table 7 (REC6.3)). As noted in the communication domain, community leaders should also be engaged (Table 4 (REC1.1)). Multidisciplinary experts who understand local contexts should be included in developing national operational plans for ending COVID-19 as a public health threat (Table 7 (REC6.1)). COVID-19 tests and treatments should be affordable for all people in all countries (Table 7 (REC6.2)).

## Discussion

Wide-ranging pandemic control measures^59–62^ have not ended COVID-19 as a public health threat^63–68^. Although this study echoes some earlier findings—for example, the Independent Panel for Pandemic Preparedness and Response^35^, the European Union 2022 communication on preparedness and response^69^ and WHO’s 2022 plan on strategic preparedness^53^—it is distinct from previous efforts^22^ given its design, which emphasized consensus building and the reporting of disagreement through the Delphi method, panellist diversity with regard to geography and disciplines, and the large sample size. The study’s focus—ending COVID-19 as a public health threat—is defined as being evidenced by the resumption of pre-pandemic social, cultural, religious, political, healthcare, economic and educational activities in each country’s context. Some retrospective matters (for example, pandemic root-cause analysis), theoretical questions and modelling were judged to be beyond the scope of the study.

Where possible, the study emphasizes recommendations that can be implemented in the short term (that is, in months, not years) to end COVID-19 as a public health threat. Although examples of countries implementing multiple recommendations exist (for example, free tests^70^, combining widespread testing and free treatment of positive cases along with digital technologies^71^, the development of vaccines providing long-lasting protection against variants^72,73^), the exceptions accentuate global challenges and provide new opportunities for action. Certain statements and recommendations resulting from this consensus process address gaps in WHO’s strategic plan^31^, most strikingly, the failure to directly address the airborne nature of transmission. Initially, the WHO incorrectly labelled airborne transmission of SARS-CoV-2 as ‘misinformation’. Only much later, after multidisciplinary scientific efforts, did the WHO recognize airborne transmission to be a predominant mode of transmission^74–76^. By contrast, this panel recommends that ‘risk communications clearly emphasize’ (Table 6 (REC4.3)) the causal link between inhalation of SARS-CoV-2 and the transmission of COVID-19 as well as policy incentivizing ‘structural prevention measures (for example, ventilation, air filtration) to mitigate airborne transmission’ (Table 6 (REC4.1)).

The WHO’s slow pace in directly addressing the airborne nature of transmission underscores why public health policy and risk communications should be based on evidence. For example, supposing that endemicity will result in lower virulence is an erroneous assumption^77–79^ that may exacerbate disproportionate risks of COVID-19 among vulnerable groups^80^. By extension, engagement with communities through effective risk communication should remain a priority for all countries.

The WHO recognizes the infodemic as a key challenge to effective communication for general populations^53,81–83^, vulnerable groups^84^ and scientists^85^. Governments, health authorities and healthcare providers should especially take care in the accuracy of their communications. The panel also emphasized that institutions should proactively monitor false health information and collaborate with trusted community leaders to refute it and enhance trust^86^.

Given the disproportionate impact that the pandemic has had on vulnerable groups to date^87–89^, the panel voiced concern that policy decisions must aim to find ways of lowering risk within these groups after resumption of the aforementioned activities (STMT6.1). As those vulnerable to COVID-19 in many countries can no longer rely on other individuals practising basic prevention measures (such as the use of face masks and isolating after testing positive), the structural changes recommended in this study (for example, indoor ventilation and filtration) assume heightened importance. Furthermore, COVID-19 continues to prompt global discussion and vigorous debate, particularly about tensions among medical ethics, civil liberties and pandemic control measures^80^. This study is no exception, with statements STMT1.6 (blaming unvaccinated individuals) and STMT3.2 (individual decisions regarding vaccination) receiving the highest levels of disagreement, underscoring the need for equitable structural interventions. In countries with widespread availability of vaccines, it is important for health authorities to distinguish between those who have clearly refused and are unlikely ever to seek vaccination and those who remain hesitant and continue to delay vaccination^90^. In the latter case, specific factors prolonging the delay can be addressed by targeted interventions. Finally, continued uncertainty about the widespread consequences of long COVID and its implications for public health policy (REC2.9) is an ongoing concern^91,92^.

Some innovations, notably vaccines^37,38^, have not been equitably distributed to low- and middle-income countries, and others, such as high-quality facemasks, have not been widely adopted in high-income countries despite their availability^93^. Some recommendations addressing pandemic inequities remain underleveraged; for example, providing more vaccines^94^ to countries with a low percentage of people vaccinated (REC6.10). Other recommendations may necessitate increased funding and time— for example, calls for continued vaccine and treatment innovations (REC2.12, REC5.2, REC5.6).

Importantly, the single significant difference in levels of panel agreement between those working in high-income countries and those working in low- and middle-income countries pertained to the role of economic incentives (REC3.6), probably reflective of sociocultural distinctions or perhaps disagreement over feasibility in implementation and ethics concerns^95,96^. Furthermore, 14% of the panellists considered themselves to be not qualified to respond to STMT4.3 concerning zoonotic variants, which probably indicates a lower understanding of biological vectors and the aetiology of variants among some of the disciplines included in the panel compared with the other topics covered^97^.

As noted above, the panellists nearly unanimously agreed on and prioritized whole-of-society and whole-of-government approaches^98–101^ (Table 8). The panellists also prioritized recommendations for communicating effectively with the public and developing technologies (for example, vaccines, therapies and services) that can reach target populations (Table 8). Failure to use these approaches risks not only prolonging COVID-19 as a public health threat, but also further diversion of resources from efforts to achieve other extant public health goals^102,103^.

### Strengths and limitations

One of the strengths of this study is its use of Delphi methodology. By demonstrating increased agreement with each subsequent round, this method enabled us to determine whether our incorporation of feedback was successful in refining the statements and recommendations, increasing the degree of consensus and, in some cases, reaching unanimity. The consistently increasing mean levels of agreement with the consensus statements and recommendations observed across all three survey rounds strengthens our confidence in the relevance of the iterative Delphi process in eliciting feedback to improve subsequent rounds. This is particularly noteworthy given that the effort to incorporate feedback from the expert panel may have resulted in more complex (for example, multiple item) statements and recommendations. Generally, there may be concerns as to the clarity of such statements; however, levels of agreement tended to be either maintained or increased, providing greater confidence in their resonance with the panel. The overall high response rates across three survey rounds speaks to both the rigorous implementation of the method and the commitment of the assembled panel of experts. Endorsement of the resultant consensus statements and recommendations by 184 organizations in 72 countries (Supplementary Table 2) at the time of publication further testifies to their global relevance.

Although the Delphi method is a robust approach (Methods) to assess levels of agreement on specific issues and explore whether a consensus can be reached, it is not without limitations. A main concern pertains to the construction of a truly representative expert panel. The sequential, multimethod sampling approach that we used (see the ‘Delphi expert panel member sample’ section in the Methods) minimized potential bias from purposive sampling of a small group and, instead, generated a large, geographically and disciplinarily diverse panel from multiple sources (that is, the core group, nominees from the core group and corresponding authors of key COVID-19 literature). While potential panellists were identified from their work related to COVID-19, infectious diseases, public health preparedness and other fields, the chairs further confirmed their appropriateness for the study by instructing them to not participate if they felt they lacked expertise concerning the pandemic. This approach appears to have been appropriate, as only 5–14% of the panellists felt they were not qualified to respond to just 5 out of the 41 statements, and 3 of the 57 recommendations. Although conducting the study in English limited the participation to English speakers, the inclusion of experts from 112 countries and territories strengthens our confidence in the potential broad applicability of these recommendations to a range of cultures and countries. With regard to the mid-study convening of the core group to discuss issues raised in the initial survey rounds, another limitation may have been that we conducted it virtually rather than in person (see the ‘Delphi data collection’ section in the Methods).

## Conclusions

The multidisciplinary panel’s emphasis on actionable, near-term recommendations guided the Delphi consensus-building process and increased the relevance of the study’s findings to a broad group of stakeholders, including governments, public health authorities, NGOs, community-based organizations, industry, and social media platforms and other media. This consensus study advances a global vision of informed decision-making on how the world can end COVID-19 as a public health threat without a return to sweeping limitations on civil liberties, without risking the health and lives of vulnerable groups, and without exacerbating economic burdens.

## Methods

### Delphi expert panel member sample

We used an iterative sampling approach to generate a large panel for this Delphi study (Fig. 1). The four co-chairs (J.V.L., A.B., A.K. and A.E.-M.) identified a core group of 40 academic, health, NGO, government and policy experts from 25 countries and territories. Selection by the co-chairs was primarily based on publication record and engagement on COVID-19 issues as well as online biographies. Twenty-nine of these experts were well known to the chairs while seven were suggested through snowball sampling to result in geographical and gender equity among the core group of 40. Furthermore, a concerted effort was made towards multidisciplinary representation in the core group, including medical sciences (such as infectious diseases, public health and vaccinology), engineering, and social sciences (such as policy, law and ethics). The core group proposed additional experts to create a global panel of approximately 400 experts. The lead chair (J.V.L.) and methodologist (D.R.) led this core group through implementation of the project. Snowball sampling was then used as core group members identified individuals with expertise in COVID-19 from their professional networks to generate an initial list of potential Delphi panel members with the goal of broad representation. In proposing experts, co-chairs focused on identifying at least one representative from at least 100 countries. One co-chair (J.V.L.) took responsibility for reviewing the suggestions, with support from a research assistant who shared recent publications and a professional biography for every proposed co-author. Many initial suggestions were of leading experts with whom the co-chairs had previously collaborated.

The core group then reviewed the panel list for under-represented countries and PubMed/Medline searches were conducted using the search term ‘COVID-19’ in combination with the names of under-represented countries to identify authors of COVID-19 research studies involving primary data collection in these countries. Authors of relevant studies were invited to participate in the Delphi panel to further increase geographical diversity and include panellists beyond the core team members’ networks. All of the panel participants were carefully vetted; most had published in one or more relevant fields.

To further validate the expertise of the panel, the study was described to the invitees (*n* = 696) with the following instructions: “If you consider your professional training and expertise applicable to the subject matter of this global consensus statement project, we encourage you to participate in the panel.” Informed consent was obtained for each panellist after explaining the purpose of the study and their expected contributions, including review and approval of the submitted manuscript, by accession to the Round 1 (R1) survey. Our objective was for invited participants to explicitly consider whether they had the necessary level of expertise before joining the Delphi panel. We do not have specific information regarding the basis of invitees’ non-participation but expect that these instructions enabled a substantial portion of non-respondents to self-select out of the study. We know that 84 invitees began the R1 survey but did not complete it; thus, if we assume that they did consider themselves to be eligible to participate but then decided not to do so, that would result in an estimated response rate of 82.1% (386 out of 470). The resultant expert panel is diverse in terms of demographic, disciplinary and geographical characteristics (Table 1).

**Table 8:** Ten highest ranked recommendations

Rank	Domain	Recommendation	Disagreement (SD+D) (%)
1	Health systems	Pandemic preparedness and response planning should adopt a whole-of-society approach that includes multiple disciplines, sectors and actors (for example, business, civil society, engineering, faith communities, mathematical modelling, military, media and psychology).	1
2	Communication	Community leaders, scientific experts and public health authorities should collaborate to develop public health messages that build and enhance individual and community trust and use the preferred means of access and communication for different populations.	0
3	Prevention	All countries should adopt a vaccines-plus approach that includes a combination of COVID-19 vaccination, prevention measures, treatment and financial incentives.	4
4	Pandemic inequities	Pandemic preparedness and response should address pre-existing social and health inequities.	1
5	Communication	Public health authorities should partner with individuals and organizations that are trusted within their communities to provide accurate, accessible information about the pandemic and inform behaviour change.	0*
6	Vaccination	Government, philanthropic and industry funding should include a focus on developing vaccines that provide long-lasting protection against multiple SARS-CoV-2 variants.	1
7	Communication	Public health professionals and authorities should combat false information proactively based on clear, direct, culturally responsive messaging that is free of unnecessary scientific jargon.	1
8	Health systems	Preparedness and response strategies should adopt whole-of-government approaches (for example, multiministry coordination) to identify, review and address resilience in health systems.	1
9	Pandemic inequities	Global trade and health organizations should coordinate with countries to negotiate the transfer of technologies enabling manufacturers in low- and middle-income countries to develop quality assured and affordable vaccines, tests and therapeutics.	1
10	Treatment and care	Promote multisectoral collaboration to accelerate the development of new therapies for all stages of COVID-19 (for example, outpatient, hospitalization and long COVID).	0*

SD+D, the combined percentage of ‘somewhat disagree’ and ‘disagree’ responses. The asterisks indicate that rounding resulted in 0% despite the presence of ≥1 response in the disagreement category.

### Delphi statement domains

The core group reviewed the published literature available up to January 2022 to draft initial statements for the first Delphi survey round, grouped in the following domains: (1) communication; (2) health systems; (3) vaccination; (4) prevention; (5) treatment and care; and (6) pandemic inequities. No formal systematic review with stringent criteria for levels of evidence was performed owing to the sheer volume of COVID-19-related published studies and the frequency at which they were and continue to be published. However, all of the authors and panellists were invited to suggest relevant papers, which were reviewed by the core group members based on journal rankings, paper citations and other metrics. In R1, panellists considered draft consensus statements based on the literature before moving to the next step of recommendations in round two (R2), which emanated from the panellists’ feedback on the statements as well as new research findings over the course of data collection from 18 February 2022 to 28 April 2022.

### Delphi method data collection

The study design consisted of digital data collection: two survey rounds (R1 and R2) of draft statements; an online consensus meeting of the core group (16 March 2022) to discuss salient issues; one round of draft recommendations (in R2); and, a final, third survey round (R3) of the consensus statements and recommendations (Fig. 1). The core group decided a priori to use a supermajority (that is, ≥67% combined agreement) minimum cut-off for consensus. This more demanding cut-off (relative to a simple majority of greater than 50%) was considered to be necessary given the project goal of supporting global policy and programmatic actions to address the COVID-19 public health crisis. We used the QualtricsXM platform to develop and distribute the surveys (round duration ranged from 1.5 to 3 weeks) with four-point Likert-type categories for measuring the level of agreement with the statements and recommendations (that is, agree, somewhat agree, somewhat disagree, disagree); a fifth ‘not qualified to respond’ option was provided given the panel’s range of COVID-19 expertise. Panellists could provide comments and suggest edits to individual statements and recommendations in text boxes, which followed each of the statements and recommendations. All rounds allowed for overall comments at the end of the survey, and the researchers reviewed 1,409, 755, and 188 comments associated with the statements in R1, R2 and R3, respectively, and 1,025 and 2,156 comments associated with the recommendations in R2 and R3, respectively. Summaries of changes based on panellist input from a previous round were available in text boxes next to each statement and recommendation in the subsequent round. Similarly, the definition for “Ending COVID-19 as a public health threat as evidenced by the resumption of social, cultural, religious, political, healthcare, economic and educational activities in each country’s context” was presented during each round so that panellists could respond to statements on the basis of a shared understanding of how the phrase “ending COVID-19 as a public health threat” was defined for the purpose of this study. In R3, panellists also ranked the top half of recommendations within each of the six domains, which were automatically randomized to mitigate order-effect bias. Using Microsoft Excel (v.16), scores were calculated and normalized using the Dowdall system to compare rankings across domains by accounting for weighting bias due to differences in the total number of recommendations in each domain^137,138^.

An important component of the data-collection process involves the discussion among core group members of issues that emerge from the early survey rounds and how best to incorporate such feedback in subsequent rounds. Given the geographical distribution of panel members and COVID-19-related travel and health concerns, we convened the core group virtually for in-depth, real-time deliberation. This web-based approach is different from in-person discussion of complicated or contentious issues; however, panel members had multiple opportunities to provide open-ended comments in the absence of dominant voices that can inhibit the expression of minority viewpoints during in-person convenings. Thus, the combination of real-time feedback (from core group members) and written feedback (from the entire panel) probably resulted in more comprehensive contributions overall.

### Delphi data analysis

Data analysis reflected the multiple-methods nature of Delphi studies and was managed by an analytic team of core group members, the study methodologist and research assistants. Across the three rounds, we ran frequencies of all statements and recommendations (Supplementary Discussion 2); the proportion who selected ‘not qualified to respond’ is reported in the data tables but removed from the denominator to calculate levels of agreement/disagreement from the relevant sample. The team then analysed the extensive qualitative data (that is, open-ended text-box comments). Specifically, comments were first reviewed individually by at least three core group members (J.V.L., co-chair; D.R., methodologist; and C.J.K.) and an additional co-author (T.M.W.). For each data collection round, comments were then discussed in online review meetings, including at least three core group members and an additional co-author. After review and discussion, comment suggestions were incorporated into statement and recommendation revisions for subsequent rounds. A supermajority of core group members (28 out of 40; 70%) participated in the online consensus meeting, which permitted in-depth breakout-group discussions on salient issues from R1 and R2 informing R3 revisions (Supplementary Discussion 3). Quantitative analysis of the final R3 results involved assigning each statement and recommendation a grade to indicate the level of combined agreement (agree + somewhat agree), using a system that has been used in other Delphi studies^139–141^ in which ‘U’ denotes unanimous (100%) agreement; ‘A’ denotes 90%–99% agreement; ‘B’ denotes 78%–89% agreement; and ‘C’ denotes 67%–77% agreement. Although all statements and recommendations exceeded the standard supermajority minimum of ≥67% combined agreement for consensus, we highlighted those with <67% for ‘agree’ alone for further analysis. Statements and recommendations were analysed using Fisher’s exact tests in Stata (v.16) to assess differences in agreement by the following panellist characteristics: income level (high income versus low- and middle-income) for country of birth and country where currently working, primary sector of employment and primary field of employment (Supplementary Discussion 2). The use of the terms combined agreement and combined disagreement are presented in the results.

### Reporting summary

Further information on research design is available in the Nature Portfolio Reporting Summary linked to this article.

## Online content

Any methods, additional references, Nature Research reporting summaries, source data, extended data, supplementary information, acknowledgements, peer review information; details of author contributions and competing interests; and statements of data and code availability are available at 10.1038/s41586-022-05398-2.

### Supplementary information

Supplementary Methods Expert panel online meeting: “Ending COVID-19 as a public health threat: consensus statement”. This file contains the minutes of the expert panel online meeting between the second and the third round of the Delphi process.

Reporting Summary

Supplementary Discussion Supplementary Discussions 1 and 2, with additional results. These quantitative results provide a broader understanding of the results presented in the main paper. Supplementary Discussion 1 contains quantitative results of agreement on statements and recommendations. Supplementary Discussion 2 contains results of bivariate analyses of the statements and recommendations by panellist characteristics.

Supplementary Table 1 The quantitative results for statements presented in rounds 1 and 2 of the Delphi process and for recommendations presented in round 2 along with a brief description of changes between rounds.

Supplementary Table 2 Institutions endorsing the statements and recommendations of ‘A multinational Delphi consensus to end the COVID-19 public health threat’.

## Figures and Tables

**Extended Data Table 1:**
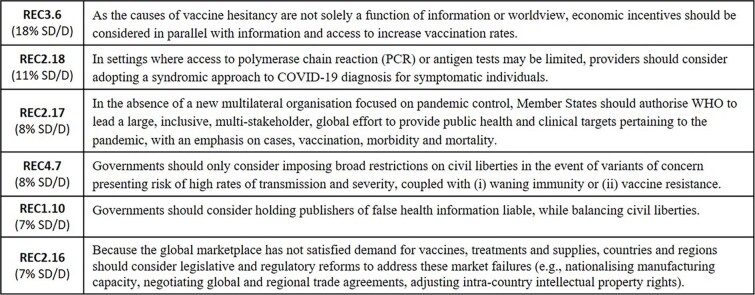
Recommendations with 5% or greater disagreement

Across the study, only six recommendations evidenced 5% or greater disagreement (combined “somewhat disagree/disagree”) among the 386 panellists. Areas of disagreement broadly included socio-political and socio-cultural considerations that vary among countries, geopolitical concerns that may impact public health guidance and distinct medical points of view.

## Data Availability

Additional data will be shared on request from the corresponding author for fair use.
