# Supplementary material for: A multinational Delphi consensus to end the COVID-19 public health threat
Source: Nature. 2022 Nov 3;611(7935):332–45. doi: 10.1038/s41586-022-05398-2 (PMC9646517; doi:10.1038/s41586-022-05398-2)
Supplement: Supplementary file 1 — Expert panel online meeting: “Ending COVID-19 as a public health threat: consensus statement”. This file contains the minutes of the expert panel online meeting between the second and the third round of the Delphi process. [file 41586_2022_5398_MOESM1_ESM.pdf]

nature portfolio

https://doi.org/10.1038/s41586-022-05398-2

Supplementary information

A multinational Delphi consensus to end the COVID-19 public health threat

In the format provided by the
authors and unedited

Supplementary Methods 1. Expert Panel Online Meeting of "Ending COVID-19 as a public health threat: consensus statement"

Date: 16 March 2022

Over two years since the COVID-19 pandemic began, individuals, communities, health systems, and entire societies continue to be impacted by it. ISGlobal initiated a three-stage Delphi study to develop a novel, action-oriented future-focused consensus statement with a set of recommendations for ending COVID-19 as a public health threat, as evidenced by the resumption of social, cultural, religious, political, healthcare, economic and educational activities in different country contexts.
As part of this study, Wilton Park and ISGlobal held a World Café dialogue for the 40 members of the consensus statement core writing group. The event followed the first and second Delphi rounds, providing the core group an opportunity to discuss issues raised in these rounds regarding the 44 consensus statements and 53 recommendations ahead of the final (third) Delphi round.

I. Introduction
By Wilton Park, Prof. Jeffrey Lazarus (consensus statement chair), and co-chairs Prof. Adeeba Kamarulzaman (morning session) and Prof. Ayman El- Mohandes (afternoon session)

II. The Delphi method
By Prof. Diana Romero
Prof. Romero explained the Delphi methodology and shared the characteristics of the 385 experts from 111 countries and territories that participated in the first Delphi round and provided an overview of the number of statements in the first and second rounds.

III. Medical ethics of patient autonomy
By Prof. Michael Parker
Prof. Parker, a bioethicist, provided insights on the topic of individual medical autonomy in relation to public health and medical ethics with the intention to provide a foundational understanding of the concept and to stimulate discussion. In the case of vaccination, he highlighted conceptual boundaries of individual autonomy (e.g., harm to self, harm to others, and burden on society).

IV. Breakout group discussions
28 experts from the core writing group were present in the two sessions.
In two groups per session, statements and recommendations were discussed that either had low levels of agreement in the first two rounds or required terminology clarifications. Specifically, seven items were discussed.
1. *Individual medical autonomy in relation to public health and medical ethics*.
Statements **3.1** and **3.2** as well as recommendation **5.8** address this issue:

- **S3.1:** Individual medical autonomy acknowledges that individuals who have decision-making capacity have the right to make decisions regarding vaccination, even when their decisions contradict their clinicians' recommendations.
- **S3.2:** When the risk of harm to others is sufficiently severe, governments may determine that the right of all individuals to good health overrides the autonomy of any one individual to choose not to be vaccinated.
- **R5.8:** Health systems should balance disease management for all population health needs, anticipating that COVID-19 patients, who are predominantly unvaccinated, cannot receive continuous, disproportionate prioritisation of care.

2. *Equitable access to vaccines & treatments*. Which demand-side interventions relative to supply-side interventions should be recommended or prioritised?
Statement **2.5** and recommendation **2.5** address this issue:

- **S2.5:** The COVID-19 pandemic continues to reveal vulnerabilities in the global supply-chain framework for essential public health supplies.
- **R2.5:** Governments and industry should engage continuous improvement disciplines for inter-country procurement, pooling and supply chain management to reduce cycle times and costs, as well as improve quality of supplies, knowledge and data to rapidly scale up the availability of medicines, vaccines and health technologies.

Prompts/Background: Continuous improvement: A set of disciplines originally initiated by Toyota and expanded over time. Two+ years into the pandemic, supply chain and transportation logistics issues, in particular, persist.

3. *Suppression of cases vs suppression of disease*.

- **General observations**: The reality is that countries are choosing quite distinct strategies for ending COVID-19 as a public health threat. Is there enough evidence for coming down on the side of one or the other? In absence of sufficient evidence, how do we err on the side of least harm? How do we acknowledge the choices of individual state actors (e.g., Brasil, Denmark, UK) in impacting global transmission, which in turn impacts on ending COVID-19 as a public health threat? Statement 5.1 addresses this issue.
- **S5.1:** For countries that have chosen suppression of disease (rather than suppression of cases) as their going-forward public health policy, health systems will require capacity for sustained treatment and long-term care.

Prompts/Background:

- Is there sufficient evidence (or even alignment among experts for an informed, educated guess) that Long COVID (as a morbidity) should be prioritised differently than other comorbidities (e.g., diabetes, obesity, etc) for ending COVID-19 as a public health threat?
- Alternatively, one might argue that Long COVID is manifesting as one (or perhaps a few) emerging comorbidity with symptoms similar to auto-immune diseases and cardiovascular risks and, therefore, addressing Long COVID should be weighted similarly to other co-morbidities in terms of the efficacy toward ending COVID-19 as a public health threat.

4. *Scope of the paper in addressing Long COVID* in relation to ending COVID-19 as a public health threat. Statements **5.2, 5.3, 5.4** and **5.5** as well as recommendations **2.1, 5.5** and **5.6** address this issue:

- **S5.2:** More effective COVID-19 therapeutic options as well as care delivery models are needed.
- **S5.3:** A standardised Long COVID case definition for children is needed.
- **S5.4:** Research is needed to determine whether infection from distinct variants of SARS-CoV-2 is associated with significant differences in long-term morbidity.
- **S5.5:** Due to insufficient evidence about Long COVID, it remains unclear how much emphasis public health policy should place on it in relation to ending COVID-19 as a public health threat.

- **R2.1:** Public health policy should take better account of the potential long-term impact of unchecked spread of COVID-19, given ongoing uncertainties about the prevalence, severity, and duration of post-COVID morbidity (aka "Long COVID")
- **R5.5:** Research funding for Long COVID to develop diagnostic tools, treatment and care, and knowledge about extrinsic factors (e.g., stigma and discrimination) should be an immediate priority.
- **R5.6:** Promote multi-sectoral collaboration and real-time information from industry for sharing clinical trial results to accelerate the development of new therapeutic options for the different stages of COVID-19 (e.g., outpatient, severe disease and Long COVID).

Prompts/Background:
We can see in other aspects of civil society that governments can - and do - regulate the flow of information (true and false information) in order to establish and maintain a balance between civil liberties (e.g., speech, religion) and public good. Examples include monitoring the flow of information about markets to mitigate stock market manipulation, criminalizing certain forms of hate speech and regulating the spectrum of what can be stated about the nutritional aspect of foods and the medical efficacy of pharmaceuticals.
The pandemic has revealed an opportunity - perhaps even a 'need' - for governments to reflect on the volume and velocity of false health information and determine whether, and, if so, how to address the risks presented by false health information.
5. *Combatting false information*. To what extent should recommendations address the intentionality of entities spreading false information and to what extent should they address the false information itself? Recommendations **1.1, 1.3, 1.6,** and **1.9** address this issue:

- **R1.1:** Governments should: (i) determine which agencies are accountable for monitoring health information; and (ii) develop monitoring tools to identify false information.
- **R1.3:** Social media companies should engage transparently with multidisciplinary researchers and developers, who are free of direct economic conflict, to create protocols and controls for their social media platforms that reduce the publication and dissemination of false health information.
- **R1.6:** Governments should actively identify and expose individuals and networks that promote false health information, and consider holding publishers of false health information liable for its adverse effects on public health, balancing their country's civil liberties.
- **R1.9:** Public health professionals and authorities should combat false information proactively based on clear, direct messaging and plain language that is free of scientific jargon.

6. *Clarification of two recommendations*.
Several other industries - and even some governments through their agencies - engage in mass-customised, tailored, targeted communications.

- "Mass-customised": Anticipating: (i) a variety of archetypes based upon various attributes (e.g., demographic, psychographic); (ii) an array of templates for communication across (iii) several means of communication.
- "Tailored": To the extent possible, adapting the mass-customized messages with individual-specific information, for example gleaned from available data about the individual.
- "Targeted": To the extent feasible, having each individual receive the message via their preferred means of communication, and, potentially, from a preferred source of information.

- **R1.4:** Community leaders and scientific experts should collaborate to develop public health messages that build and enhance community and individual trust and utilise each member of the general public's preferred means of access and communication.
- **R3.4:** To combat vaccine hesitancy, mass-customised, tailored messages that address reflect the underlying bases of an individual's concerns should be utilised in targeted public health communications.

7. *Drafting recommendations*.

Questions for consideration based upon observers and contributors to this process who come from disciplines other than public health:
Do public health practitioners, generally, have a bias toward **recommending centralization** (as distinct from decentralization) within governments? If so, what opportunities may not be

fully considered when not debating or recommending decentralized approaches to pandemic response?
And do public health practitioners, generally, have a **bias toward recommending institutionalization** (e.g., creation or reliance on NGOs) for social engagement, as distinct from direct engagement with grassroots efforts within communities (e.g., the seemingly spontaneous 'rise' of neighborhood and community-based mutual aid efforts that often times acted 'faster' than government, industry or NGO responses). If so, what opportunities may not be fully considered when not recognizing the historic role of mutual aid efforts, community organizers and social movements?

- **General observations**: Target audiences with sufficient specificity that the recommendations are actionable
- **General observations**: Identify specific areas of the pandemic that need particular focus for scoping, narrative & systematic reviews towards ending COVID-19 as a public health threat.
- **General observations**: *Degree of centralization*. There seems to be a tendency in public health to recommend government centralization as a primary orientation for response to health crises.
- **General observations**: *Reliance on institutionalization*. Similarly, there seems to be a primary orientation to assume institutionalization as a response to crises. For instance, edits in the consensus statements 'away' from social movements and 'toward' civil society or NGOs. That said, we have significant experiential evidence of the important role informal and semi-formal mobilizing, organizing and even manufacturing has played throughout the pandemic. There seems to be an assumption to discount the potential impact of grassroots and community organizers.
- **Potential new recommendation**: Emulate the experience of the Open Source and White Hat (Ethical) Hacking communities by cultivating and supporting a community of 'white hats' for exposing and addressing hot-spots and networks disseminating false information, with immediate emphasis on mal-information.

V. Closing remarks and next steps

Report prepared by Prof. Jeffrey V. Lazarus and Wilton Park
