# Supplementary material for: A multinational Delphi consensus to end the COVID-19 public health threat
Source: Nature. 2022 Nov 3;611(7935):332–45. doi: 10.1038/s41586-022-05398-2 (PMC9646517; doi:10.1038/s41586-022-05398-2)
Supplement: Supplementary file 2 — Reporting Summary [file 41586_2022_5398_MOESM2_ESM.pdf]

nature portfolio

Corresponding author(s): Jeffrey V. Lazarus

Last updated by author(s): Sep 2, 2022

Reporting Summary

Nature Portfolio wishes to improve the reproducibility of the work that we publish. This form provides structure for consistency and transparency in reporting. For further information on Nature Portfolio policies, see our Editorial Policies and the Editorial Policy Checklist.

Statistics

For all statistical analyses, confirm that the following items are present in the figure legend, table legend, main text, or Methods section.

n/a	Confirmed	
☐	☒	The exact sample size (*n*) for each experimental group/condition, given as a discrete number and unit of measurement
☐	☒	A statement on whether measurements were taken from distinct samples or whether the same sample was measured repeatedly
☐	☒	The statistical test(s) used AND whether they are one- or two-sided<br>*Only common tests should be described solely by name; describe more complex techniques in the Methods section.*
☒	☐	A description of all covariates tested
☒	☐	A description of any assumptions or corrections, such as tests of normality and adjustment for multiple comparisons
☐	☒	A full description of the statistical parameters including central tendency (e.g. means) or other basic estimates (e.g. regression coefficient) AND variation (e.g. standard deviation) or associated estimates of uncertainty (e.g. confidence intervals)
☐	☒	For null hypothesis testing, the test statistic (e.g. *F*, *t*, *r*) with confidence intervals, effect sizes, degrees of freedom and *P* value noted<br>*Give P values as exact values whenever suitable.*
☒	☐	For Bayesian analysis, information on the choice of priors and Markov chain Monte Carlo settings
☒	☐	For hierarchical and complex designs, identification of the appropriate level for tests and full reporting of outcomes
☒	☐	Estimates of effect sizes (e.g. Cohen's *d*, Pearson's *r*), indicating how they were calculated

Our web collection on statistics for biologists contains articles on many of the points above.

Software and code

Policy information about availability of computer code

Data collection	QualtricsXM® platform
Data analysis	Microsoft Excel v16 and Stata v16

For manuscripts utilizing custom algorithms or software that are central to the research but not yet described in published literature, software must be made available to editors and reviewers. We strongly encourage code deposition in a community repository (e.g. GitHub). See the Nature Portfolio guidelines for submitting code & software for further information.

Data

Policy information about availability of data

All manuscripts must include a data availability statement. This statement should provide the following information, where applicable:

- Accession codes, unique identifiers, or web links for publicly available datasets
- A description of any restrictions on data availability
- For clinical datasets or third party data, please ensure that the statement adheres to our policy

Additional data will be shared upon request from the corresponding author for fair use.

Human research participants

Policy information about studies involving human research participants and Sex and Gender in Research.

Reporting on sex and gender	We report proportion of participants selecting "man", "woman", "non-binary or gender diverse", and "prefer not to say". As neither sex nor gender are determinative of one's subject matter expertise (the primary inclusion criteria for the panel), we did not consider any tests to investigate differences in this expertise by gender. Sex was not collected. The panel reported near-gender parity between men and women.
Population characteristics	The panel's characteristics are reported in Table 4 and are as follows:<br><br>Gender: Man (58%), Woman (40%), No response (2%)<br><br>Primary sector of employment: Civil Society (66%), Private (16%), Academic (10%), Public (5%), Other (2%), No response (1%)<br><br>Primary field of employment: Public Health (41%), Clinical Research/Care (24%), Health Policy/Advocacy (17%), Basic/Physical/Mathematical Sciences (11%), Other (6%), No response (2%)<br><br>Country income level: Low or middle income country (50%), High income country (48%), No response (1%)<br><br>Global region of origin: Europe & Central Asia (30%), Latin America & Caribbean (15%), East Asia & Pacific (13%), North America (12%), Sub-Saharan Africa (11%), Middle East & North Africa (9%), South Asia (9%)
Recruitment	We employed an iterative sampling approach to generate a large panel for this Delphi study (Figure 1). The four co-chairs (JVL, AB, AK, AE-M) identified a core group of 40 experts from 25 countries, representing discipline, geographic and gender diversity to guide development of consensus statements and recommendations to end COVID-19 as a public health threat. The lead chair (JVL) and methodologist (DR) led this core group through implementation of the project. Snowball sampling was then used as core group members identified individuals with expertise in COVID-19 from their professional networks to generate an initial list of potential Delphi panel members with the goal of broad representation. The core group reviewed the panel list for under-represented countries and PubMed/Medline searches were conducted using the search term "COVID-19" in combination with the names of under-represented countries to identify authors of COVID-19 research studies involving primary data collection in these countries. Authors of relevant studies were invited to participate in the Delphi panel in order to further increase geographic diversity and include panellists beyond the core team members' networks. To further validate the expertise of the panel, the study was described to the invitees, who were instructed not to participate if they considered themselves lacking the necessary level of expertise. Informed consent was obtained for each panellist after explaining the purpose of the study and their expected contributions, including review and approval of the submitted manuscript, by accession to the R1 survey.
Ethics oversight	This study has been exempted from ethical review by the Research Ethics Committee of the Hospital Clinic, University of Barcelona, Spain.

Note that full information on the approval of the study protocol must also be provided in the manuscript.

Field-specific reporting

Please select the one below that is the best fit for your research. If you are not sure, read the appropriate sections before making your selection.

☐ Life sciences ☒ Behavioural & social sciences ☐ Ecological, evolutionary & environmental sciences

For a reference copy of the document with all sections, see nature.com/documents/nr-reporting-summary-flat.pdf

Behavioural & social sciences study design

All studies must disclose on these points even when the disclosure is negative.

Study description	This is a three-round, standard Delphi consensus statement study.
Research sample	Selection of participants was primarily based on publication record and engagement on the issues, not necessarily to be representative of any country, sector, or field. A number of these experts were well known to the chairs while others were suggested through snowball sampling to ensure broad international geographic and gender equity among the core group of 40. Further, a concerted effort was made to ensure multi-disciplinary representation in the core group, including medical sciences (e.g., infectious diseases, and public health, vaccinology), engineering, and social sciences (e.g., policy, law, ethics). It was a prerequisite that the core group would be able to suggest additional experts to create the global panel of 386 experts (58% male, 66% working primarily in civil society, 41% working primarily in public health, and 50% working in low and middle income countries).
Sampling strategy	We employed an iterative sampling approach via a core group of 40 public health experts that represented gender and geographic

Sampling strategy	parity in order to create a robust international multi-disciplinary panel that could substantively engage with the content of the study.
Data collection	We used the QualtricsXM® platform to develop and distribute the surveys (round duration ranged from 1.5 to 3 weeks) with 4-point Likert-type categories for measuring level of agreement with the statements and recommendations (i.e., Agree, Somewhat agree, Somewhat disagree, Disagree); a fifth 'not qualified to respond' option was provided given the panel´s range of COVID-19 expertise. Panellists could provide comments and suggest edits to individual statements and recommendations in text boxes, which followed each of the statements and recommendations.
Timing	The study design consisted of digital data collection: two survey rounds (R1 and R2) of draft statements and recommendations (18 February-15 March 2022); an online consensus meeting of the core group (16 March 2022); and a third survey round (R3) (15 April-28 April 2022).
Data exclusions	No data were excluded.
Non-participation	All co-authors participated in the Delphi process and approved the submitted consensus statement manuscript.
Randomization	Panelists participated as a single group. However, when ranking the recommendations, the recommendation items were randomized within each domain so as to avoid question-order bias.

Reporting for specific materials, systems and methods

We require information from authors about some types of materials, experimental systems and methods used in many studies. Here, indicate whether each material, system or method listed is relevant to your study. If you are not sure if a list item applies to your research, read the appropriate section before selecting a response.

Materials & experimental systems

n/a	Involved in the study
☒	☐ Antibodies
☒	☐ Eukaryotic cell lines
☒	☐ Palaeontology and archaeology
☒	☐ Animals and other organisms
☒	☐ Clinical data
☒	☐ Dual use research of concern

Methods

n/a	Involved in the study
☒	☐ ChIP-seq
☒	☐ Flow cytometry
☒	☐ MRI-based neuroimaging
