# Supplementary material for: A multinational Delphi consensus to end the COVID-19 public health threat
Source: Nature. 2022 Nov 3;611(7935):332–45. doi: 10.1038/s41586-022-05398-2 (PMC9646517; doi:10.1038/s41586-022-05398-2)
Supplement: Supplementary file 3 — Supplementary Discussions 1 and 2, with additional results. These quantitative results provide a broader understanding of the results presented in the main paper. Supplementary Discussion 1 contains quantitative results of agreement on statements and recommendations. Supplementary Discussion 2 contains results of bivariate analyses of the statements and recommendations by panellist characteristics. [file 41586_2022_5398_MOESM3_ESM.pdf]

Supplementary Discussion 1: Quantitative results of agreement on statements and recommendations

Thirty-nine of 41 (95%) *statements* across all domains scored a grade of A (90-99% of combined agreement; “agree” + “somewhat agree”), with the exception of one statement on communication (STMT1.6) and one on vaccination (STMT3.2) that scored a grade of B (78%–89% combined agreement). The percentages of combined agreement across all statements ranged from 84% (STMT3.2) to 99.7% (STMT5.3). Based on “ agree” alone (without “somewhat agree”) seven statements scored at or above 90%: STMT1.4, STMT2.6, STMT4.1, STMT5.2, STMT5.3, S5.4 and S6.1. The percentages of combined disagreement (“disagree” + “somewhat disagree”) ranged from 16% (STMT3.2) to 0% (STMT5.3).

Likewise, for the *recommendations* almost all (53 of 57; 93%) scored a grade of A, except for two recommendations scoring B, on health systems (REC2.18) and vaccination (REC3.6), respectively, and two recommendations on communications (REC1.1) and treatment and care (REC5.1), respectively, that reached unanimous agreement (100%). Across the 57 recommendations, combined agreement ranged from a low of 82% (REC3.6) to a high of 100% (REC1.1 and REC5.1). Over half of the recommendations scored at or above 90% based on “agree” alone. Combined disagreement ranged from a low of 0% (REC1.1 and REC5.1) to a high of 18% (REC3.6).

Communicate effectively

Six of the seven statements on communication scored a grade of A (90-99% of combined agreement), with one statement (STMT1.6) scoring a grade of B. The statement about combating false information (STMT1.4) was the only communication statement that scored at or above 90% based upon “agree” alone. Nine of the communication recommendations scored a grade of A, and one had unanimous agreement (REC1.1). The percentage of “agree” alone ranged from 57% (STMT1.6) to 94% (STMT1.7) for the statements, and 76% (REC1.10) to 96% (REC1.1) for the recommendations. The combined disagreement percent ranged from 1% (STMT1.1, STMT1.4, STMT1.7) to 12% (STMT1.6) for the statements and 0% (REC1.1) to 7% (REC1.10) for the recommendations.

Strengthen health systems

All nine statements on health systems had a grade of A. Of the 18 recommendations 17 scored A, and one scored B (REC2.18). The percentage of combined agreement ranged from 91% (STMT2.1) to 99% (STMT2.6 and STMT2.8) for the statements and 89% (STMT2.18) to 100% (REC2.1) for the recommendations. One statement (STMT2.6) and eight recommendations (REC2.1, REC2.2, REC2.4-REC2.7, REC2.10, REC2.11) had a score of 90% or above based on “agree” alone. The level of combined disagreement ranged from 1% (STMT2.6) to 9% (STMT2.1) for the statements and from 0% (REC2.1-REC2.3) to 11% (REC2.18) for the recommendations.

Emphasise vaccination

Of the six statements in this domain, five were at grade ‘A’ consensus with S3.2 receiving a ‘B’ grade. Five of the six recommendations had an ‘A’ grade, with R3.6 receiving a ‘B’ grade. No statement scored a 90% or higher solely on “agree” alone, whereas all recommendations but REC3.6 did. The level of combined disagreement ranged from 4% (STMT3.3, STMT3.6) to 16% (STMT3.2) in the statements and 0% (REC3.1) to 18% (REC3.6) in the recommendations.

Promote preventive behaviours

All seven statements scored an A grade. One statement (STMT4.1) scored 90% or above based on “agree” alone responses. Similarly, all seven recommendations scored an A grade, and REC4.3 scored 90% or above for “agree” alone. Combined disagreement levels among the statements ranged from 0% (STMT4.1) to 7% (STMT4.7), and from 1% (REC4.1) to 8% (REC4.7) among the recommendations.

Expand treatments
The four statements on treatment and care all received a grade of A. Five recommendations had a grade of A (REC5.3-REC5.6) and REC5.1 scored a grade of U. The percentage of combined agreement ranged from 92% (STMT5.1) to 100% (STMT5.3) for the statements and from 97% (REC5.6) to 100% (REC5.1, REC5.2, REC5.6) for the recommendations. Three of the statements (STMT5.2-STMT5.4) and four of the recommendations (REC5.1-REC5.4) had a grade of A based on “agree” alone. For the statements, the level of combined disagreement ranged from 0% (STMT5.3) to 7% (STMT5.1) and for the recommendations from 0% (REC5.1, REC5.2, REC5.3) to 3% (REC5.6).

Eliminate inequities
All statements concerning pandemic inequities scored a grade of A. STMT6.1 is the only to score at or above 90% based upon “agree” alone. Similarly, all ten of the pandemic inequities recommendations scored a grade of A. All but three recommendations (REC6.8-REC6.10) scored at or above 90% for “agree” only. The percentage of combined agreement for the statements ranged from 90% (STMT6.4) to 98% (STMT6.1, STMT6.6), and 98% (REC6.10) to 100% (REC6.1) for the recommendations. The level of combined disagreement ranged from 2% (STMT6.1, TMTS6.3, STMT6.6) to 10% (STMT6.4) for the statements and from 0% (REC6.1-REC6.4) to 2% (REC6.9, REC6.10) for the recommendations.

Supplementary Discussion 2. Results of bivariate analyses of the statements and recommendations by panellist characteristics

Each statement and recommendation was analysed using Fisher's exact test to assess differences in disagreement by the following sample characteristics: income level (high-income [HIC] vs. low- and middle-income [LMIC]), country of birth and country where currently working, primary sector of employment, and primary field of employment. We decided to collect the demographic data a priori as these variables are where we hypothesized there might be differences in approaches to addressing the COVID-19 public health threat. Specific bivariate analyses were then carried out based on our review of the data identifying statements and recommendations with relatively lower levels of agreement, in an effort to determine the potential bases for greater disagreement. We report significant differences at the $p<0.05$ level.

Among panellists who disagreed (Somewhat disagree [SD] and Disagree [D] combined; Table 1) with the statements and recommendations, significant differences were found such that those working in LMIC were more likely to disagree than those working in HIC for:

- (STMT1.2) Public health authorities contribute to the dissemination of false information when their communications do not reflect current scientific understanding that transmission of SARS-Cov-2 is primarily airborne (p=0.032).
- (STMT1.3) Governments have inconsistently counteracted false information in the context of the COVID-19 pandemic (p=0.004). (This was also significant among those *born* in LMIC vs HIC; p=0.028.)
- (STMT2.1) The world has not implemented an evidence-based, globally agreed-upon set of minimum COVID-19 pandemic response standards addressing monitoring, prevention, treatment, and care (p=0.003). (This was also significant among those *born* in LMIC vs HIC; p=0.023.)
- (STMT2.3) Health systems are continuing to face abnormal staffing shortages due to the mental and physical health impacts on their workers from the COVID-19 pandemic (p=0.021).
- (STMT3.5) Continued low levels of trust in information from government sources are associated with vaccine hesitancy (p=0.003).
- (STMT6.6) The global pandemic response has generally not taken into account the underlying role of social determinants of health (p=0.046).
- (REC4.5) All countries should adopt a "vaccines plus" approach that includes a combination of COVID-19 vaccination, prevention measures, treatment and financial incentives (p=0.027).

Many of these statements are critical of implementers of COVID-19 pandemic response efforts and may reflect disagreement in how LMIC vs HIC handled the response in their country. The difference for recommendation towards a "vaccines plus" approach may be appreciably explained by global vaccine inequity that resulted in a limited capacity for LMIC to implement any approach with vaccination as their foundational strategy at the time this study was implemented.

Significant differences between primary sector and field of employment were also found among panellists reporting disagreement for:

- (STMT1.1) The volume and velocity of information during the COVID-19 pandemic have made it difficult for people to assess the accuracy of information. (Those disagreeing are more likely to be working in health policy than other fields; p=0.046.)
- (STMT1.3) Governments have inconsistently counteracted false information in the context of the COVID-19 pandemic. (Those primarily working in academia or the public sector are more likely to disagree than civil society and the private sector; p=0.006.)
- (REC2.16) Because the global marketplace has not satisfied demand for vaccines, treatments and supplies, countries and regions should consider legislative and

regulatory reforms to address these market failures (e.g., nationalising manufacturing capacity, negotiating global and regional trade agreements, adjusting intra-country intellectual property rights). (Among those disagreeing, they are more likely to be working in the academic sector; p=0.050.)

For one recommendation, those reporting disagreement were more likely to be working in a HIC than in a LMIC:

- (REC5.5) Governments should now prioritise early case detection so that health systems can facilitate earlier treatment and care (p=0.018).
