# Supplementary material for: A multinational Delphi consensus to end the COVID-19 public health threat
Source: Nature. 2022 Nov 3;611(7935):332–45. doi: 10.1038/s41586-022-05398-2 (PMC9646517; doi:10.1038/s41586-022-05398-2)
Supplement: Supplementary file 5 — Institutions endorsing the statements and recommendations of ‘A multinational Delphi consensus to end the COVID-19 public health threat’. [file 41586_2022_5398_MOESM5_ESM.pdf]

**Supplementary Table 2. Institutions endorsing the statements and recommendations of "A multinational Delphi consensus to end the COVID-19 public health threat"**

| Institution | City | Country |
|---|---|---|
| Academia Nacional de Ciencias de Costa Rica | San José | Costa Rica |
| Africa Advocacy Foundation | London | United Kingdom of Great Britain and Northern Ireland |
| Africa Canada Artificial Intelligence and Data Innovation Consortium (ACADIC) | Toronto | Canada |
| Africa Centres for Disease Control and Prevention, African Union Commission | Addis Ababa | Ethiopia |
| African Center for Global Health and Social Transformation (ACHEST) | Kampala | Uganda |
| African Population and Health Research Center | Nairobi | Kenya |
| Aga Khan University | Karachi | Pakistan |
| AIDS Society of India | Mumbai | India |
| Ain Shams University | Cairo | Egypt |
| Alexandria University | Alexandria | Egypt |
| Alianza Latinoamericana de Salud Global (ALASAG) | Santiago | Chile |
| American Association for Aerosol Research (AAAR) | Reston | United States of America |
| Ankara Yildirim Beyazit University, Ankara City Hospital | Ankara | Turkey |
| Ashoka University | Sonepat | India |
| Asociación Latinoamericana para el Estudio del Hígado (ALEH) | Santiago | Chile |
| Baku Health Center | Baku | Azerbaijan |
| Biomedical Research and Therapeutic Vaccines Institute | Ciudad Bolívar | Bolivarian Republic of Venezuela |
| Bitran y Asociados | Santiago | Chile |
| British Columbia Centre for Excellence in HIV/AIDS | Vancouver | Canada |
| Burnet Institute | Melbourne | Australia |
| Canadian Aerosol Transmission Coalition | Ottawa | Canada |
| Canadian Association of PPE Manufacturers | Cambridge | Canada |
| Center for Outcomes Research in Liver Diseases (COR-LD) | Washington, D.C. | United States of America |
| Centre for Digital Therapeutics | Toronto | Canada |

| | | |
|---|---|---|
| Centre for Infectious Disease Research in Zambia (CIDRZ) | Lusaka | Zambia |
| Centre for the AIDS Programme of Research in South Africa (CAPRISA) | Durban | South Africa |
| Centro de Estudos e Pesquisas de Direito Sanitário da Faculdade de Saúde Pública da USP | São Paulo | Brazil |
| Centro de Investigaciones del Mar y la Atmósfera (CIMA, UBA-CONICET) | Buenos Aires | Argentina |
| Child Health Research Foundation | Dhaka | Bangladesh |
| Chinese Center for Disease Control and Prevention | Beijing | China |
| Chulalongkorn University | Bangkok | Thailand |
| Clinical Hospital Center of Rijeka | Rijeka | Croatia |
| Coalition for Community & Healthcare Acquired Infection Reduction (CHAIR) | Cambridge | Canada |
| *Communications Medicine* | London | United Kingdom of Great Britain and Northern Ireland |
| CONVINCE USA | New York City | United States of America |
| Council on Health Research for Development (COHRED) | Geneva | Switzerland |
| Crookston Law | Toronto | Canada |
| Dar Al Uloom University | Riyadh | Saudi Arabia |
| Data Covid19 Chile | Santiago | Chile |
| Department of Medical Sciences of the Academy of Sciences and Arts of Bosnia and Herzegovina | Sarajevo | Bosnia and Herzegovina |
| Digestive Disease Research Institute, Tehran University of Medical Sciences | Tehran | Iran |
| Directorate of Health Services in Kashmir | Kashmir | India |
| Djilali Bounaama Khemis Miliana University | Khemis Miliana | Algeria |
| Dr. Rajendra Prasad Government Medical College | Kangra at Tanda | India |
| Effective Basic Services (eBASE) Africa | Bamenda | Cameroon |
| Emory University School of Medicine | Atlanta | United States of America |
| EndCoronavirus.org | Cambridge | United States of America |
| Erasmus University Medical Center | Rotterdam | Netherlands |
| European Association for the Study of the Liver (EASL) | Geneva | Switzerland |

| | | |
|---|---|---|
| European Federation of National Organisations Working with the Homeless (FEANTSA) | Brussels | Belgium |
| European Public Health Alliance | Brussels | Belgium |
| European Public Health Association (EUPHA) | Utrecht | Netherlands |
| European Society for Primary Care Gastroenterology (ESPCG) | Stockholm | Sweden |
| Fiocruz Amazonia | Manaus | Brazil |
| Fondation Congolaise pour la Recherche Médicale | Brazzaville | Republic of the Congo |
| Fundación Plenitud | Santo Domingo | Dominican Republic |
| Gamaleya National Research Center for Epidemiology and Microbiology of the Russian Ministry of Health | Moscow | Russian Federation |
| Generations for Health | Madrid | Spain |
| Global Health Literacy Academy | Risskov | Denmark |
| Gobierna Consulting Firm | Lima | Peru |
| Health and Sustainable Development Foundation | Yilan City | Taiwan |
| Icahn School of Medicine at Mount Sinai (ISMMS) | New York City | United States of America |
| Ifakara Health Institute | Dar es Salaam | United Republic of Tanzania |
| Independent Lebanese Committee for the Elimination of COVID-19 | Beirut | Lebanon |
| Indraprastha Institute of Information Technology Delhi (IIIT-Delhi) | New Delhi | India |
| Institut de Recherche en Santé de Surveillance Epidemiologique et de Formation (IRESSEF) | Dakar | Senegal |
| Institut de Recherche en Sciences de la Santé - Clinical Research Unit of Nanoro (IRSS-CRUN) | Nanoro | Burkina Faso |
| Institute for the Advancement of Health & Well-being | Princeton | United States of America |
| Institute of Global Health, Université de Genève | Geneva | Switzerland |
| Instituto #SaludsinBulos | Madrid | Spain |
| Instituto de Salud Global de Barcelona (ISGlobal) | Barcelona | Spain |
| Instituto de Saúde Coletiva - Federal University of Bahia | Salvador | Brazil |
| Instituto Leônidas & Maria Deane | Manaus | Brazil |
| Instituto Nacional de Ciencias Médicas y Nutrición Salvador Zubirán | México D.F. | Mexico |

| | | |
|---|---|---|
| Instituto Oswaldo Cruz - Fundação Oswaldo Cruz | Rio de Janeiro | Brazil |
| International Centre for Research on Women (ICRW) | Washington, D.C. | United States of America |
| International Livestock Research Institute (ILRI) | Nairobi | Kenya |
| International Medical University | Kuala Lumpur | Malaysia |
| Izmir Katip Celebi University Ataturk Research and Training Hospital Infectious Disease Department | Izmir | Turkey |
| Kabul University of Medical Sciences | Kabul | Afghanistan |
| Kenya Medical Research Institute | Nairobi | Kenya |
| KHANA Center for Population Health Research | Phnom Penh | Cambodia |
| Kirby Institute, Unversity of New South Wales (UNSW) Sydney | Sydney | Australia |
| Koç University İşbank Center for Infectious Diseases (KUISCID) | Istanbul | Turkey |
| Kwame Nkrumah University of Science and Technology School of Public Health | Kumasi | Ghana |
| Makerere Univerity College of Health Sciences | Kampala | Uganda |
| Makerere University Lung Institute | Kampala | Uganda |
| Mälardalen University | Västerås | Sweden |
| Management Sciences for Health | Medford | United States of America |
| Mi-Health Europe | Amsterdam | Netherlands |
| Ministry of Health of the Republic of Slovenia | Ljubljana | Slovenia |
| Mogadishu University | Mogadishu | Somalia |
| National Aerospace University "Kharkiv Aviation Institute" | Kharkiv | Ukraine |
| National and Kapodistrian University of Athens | Athens | Greece |
| National and Kapodistrian University of Athens Medical School | Athens | Greece |
| National Center for Disease Control and Public Health, The University of Georgia | Tbilisi | Georgia |
| National Centre for Infectious Diseases | Singapore | Singapore |
| National Health Care for the Homeless Council | Nashville | United States of America |
| *Nature Cardiovascular Research* | New York City | United States of America |

| | | |
|---|---|---|
| *Nature Communications* | London | United Kingdom of Great Britain and Northern Ireland |
| *Nature Medicine* | New York City | United States of America |
| *Nature Metabolism* | Berlin | Germany |
| *Nature Reviews Cardiology* | London | United Kingdom of Great Britain and Northern Ireland |
| *Nature Reviews Clinical Oncology* | London | United Kingdom of Great Britain and Northern Ireland |
| *Nature Reviews Disease Primers* | London | United Kingdom of Great Britain and Northern Ireland |
| *Nature Reviews Endocrinology* | London | United Kingdom of Great Britain and Northern Ireland |
| *Nature Reviews Gastroenterology & Hepatology* | London | United Kingdom of Great Britain and Northern Ireland |
| *Nature Reviews Nephrology* | London | United Kingdom of Great Britain and Northern Ireland |
| *Nature Reviews Neurology* | London | United Kingdom of Great Britain and Northern Ireland |
| *Nature Reviews Rheumatology* | London | United Kingdom of Great Britain and Northern Ireland |
| *Nature Reviews Urology* | London | United Kingdom of Great Britain and Northern Ireland |
| New England Complex Systems Institute (NECSI) | Cambridge | United States of America |
| *npj Digital Medicine* | New York City | United States of America |
| O'Neill Institute for National and Global Health Law, Georgetown University | Washington, D.C. | United States of America |
| Observatório COVID-19 BR | São Paulo | Brazil |
| ONOM Foundation | Ulaanbaatar | Mongolia |
| OzSAGE | Sydney | Australia |
| Paris Dauphine University - PSL | Paris | France |
| Peking University | Beijing | China |
| Programa de Estudio y Control de Enfermedades Tropicales (PECET), Universidad de Antioquia | Medellin | Colombia |

| | | |
|---|---|---|
| Protect Their Future | | United States of America |
| Public Health Forum | Ramat Gan | Israel |
| Rede Análise | Porto Alegre | Brazil |
| Rede de Pesquisa Solidária em Políticas Públicas & Sociedade | São Paulo | Brazil |
| Research Institute of Virology of the Republican Specialized Scientific and Practical Medical Center for Epidemiology, Microbiology, Infectious and Parasitic Diseases | Tashkent | Uzbekistan |
| Saw Swee Hock School of Public Health, National University of Singapore | Singapore | Singapore |
| School of Medicine and Pharmacy, University Mohammed V | Rabat | Morocco |
| School of Medicine, Climate & Health Program, University of Colorado | Aurora | United States of America |
| School of Public Health, University of Zambia | Lusaka | Zambia |
| SHARE Global Health Foundation | Amsterdam | Netherlands |
| Smart Phases | Plattsburgh | United States of America |
| Society on Liver Disease in Africa (SOLDA) | Utrecht | Netherlands |
| Swiss Academies of Arts and Sciences | Berne | Switzerland |
| Swiss Tropical and Public Health Institue (Swiss TPH) | Basel | Switzerland |
| Syrian Private University | Damascus | Syrian Arab Republic |
| Tashkent Pediatric Medical Institute | Tashkent | Uzbekistan |
| Team Airborne UK | London | United Kingdom of Great Britain and Northern Ireland |
| Tennessee State University | Chattanooga | United States of America |
| The Institute of Applied Sciences (INES) Ruhengeri | Musanze | Rwanda |
| The Royal Hospital | Muscat | Oman |
| Tohoku University Graduate School of Medicine | Sendai | Japan |
| Transmissible Public Health Learning Solutions | Houten | Netherlands |
| Two Oceans in Health | Santo Domingo | Dominican Republic |
| UNITE Network | Lisbon | Portugal |
| Universal Health Monitor | Potomac | United States of America |
| Universidad de Costa Rica | San José | Costa Rica |

| | | |
|---|---|---|
| Universidad de San Carlos de Guatemala | Guatemala | Guatemala |
| Universidad del Desarrollo | Santiago | Chile |
| Universidad Nacional de Hurlingham | Hurlingham | Argentina |
| Universidad Nacional de Tres de Febrero | Caseros | Argentina |
| Università Cattolica del Sacro Cuore | Rome | Italy |
| Universitat Internacional de Catalunya | Barcelona | Spain |
| University Medical Centre Ljubljana | Ljubljana | Slovenia |
| University of Bucharest | Bucharest | Romania |
| University of Cabo Verde (Uni-CV) | Praia | Cape Verde |
| University of California San Francisco (UCSF) | San Francisco | United States of America |
| University of Chakwal | Chakwal | Pakistan |
| University of Gothenburg | Gothenburg | Sweden |
| University of Health and Allied Sciences | Ho | Ghana |
| University of Malaya | Kuala Lumpur | Malaysia |
| University of Southern Denmark and Odense University Hospital | Odense | Denmark |
| University of the Americas | Santiago | Chile |
| University of the Witwatersrand | Johannesburg | South Africa |
| University of Toronto | Toronto | Canada |
| University of Valencia | Valencia | Spain |
| University of Vlore ”Ismail Qemali” | Vlore | Albania |
| University of Zambia | Lusaka | Zambia |
| University Ss. Cyril and Methodius | Skopje | Republic of North Macedonia |
| University Teaching Hospital | Butare | Rwanda |
| University Ziane Achour | Djelfa | Algeria |
| Vaccine Innovation Center, Korea University College of Medicine | Seoul | South Korea |
| Victorian Department of Health | Melbourne | Australia |
| Vietnam One Health University Network | Pham Duc Phuc | Viet Nam |
| Wits Reproductive Health and HIV Institute (Wits RHI) | Johannesburg | South Africa |
| Women in Global Health | Washington, D.C. | United States of America |
| World Federation of Public Health Associations | Geneva | Switzerland |
| World Health Network | Cambridge | United States of America |
| Yong Loo Lin School of Medicine, National University of Singapore | Singapore | Singapore |

| | | |
|---|---|---|
| Zero Covid Canada | Ottawa | Canada |
| Zero Covid US | Seattle | United States of America |
| Zero COVID-19 | Nashville | United States of America |
| ZeroCovid Colombia | Bogotá | Colombia |
| ZeroCovid Germany | Berlin | Germany |
| ZeroCovid Iberoamerica | | |
